# Analysis of multi-strain infection of vaccinated and recovered population through epidemic model: Application to COVID-19

**DOI:** 10.1371/journal.pone.0271446

**Published:** 2022-07-29

**Authors:** Olusegun Michael Otunuga

**Affiliations:** Department of Mathematics, Augusta University, Augusta, GA, United States of America; Hodeidah University, YEMEN

## Abstract

In this work, an innovative multi-strain *SV EAIR* epidemic model is developed for the study of the spread of a multi-strain infectious disease in a population infected by mutations of the disease. The population is assumed to be completely susceptible to *n* different variants of the disease, and those who are vaccinated and recovered from a specific strain *k* (*k* ≤ *n*) are immune to previous and present strains *j* = 1, 2, ⋯, *k*, but can still be infected by newer emerging strains *j* = *k* + 1, *k* + 2, ⋯, *n*. The model is designed to simulate the emergence and dissemination of viral strains. All the equilibrium points of the system are calculated and the conditions for existence and global stability of these points are investigated and used to answer the question as to whether it is possible for the population to have an endemic with more than one strain. An interesting result that shows that a strain with a reproduction number greater than one can still die out on the long run if a newer emerging strain has a greater reproduction number is verified numerically. The effect of vaccines on the population is also analyzed and a bound for the herd immunity threshold is calculated. The validity of the work done is verified through numerical simulations by applying the proposed model and strategy to analyze the multi-strains of the COVID-19 virus, in particular, the Delta and the Omicron variants, in the United State.

## 1 Introduction

The growing threat of infectious diseases with resistance to drugs and vaccinations, causing large number of deaths worldwide, is a cause for concern to the medical community and the general population. Scientists around the world are working to learn more about such diseases in order to study how likely an emerging variant of the disease can spread more easily than existing original variants. More data and analyses are needed for such study. These analyses will shed more light on the possibility of reinfections in people who already recovered from original strain, and infections in people who are fully vaccinated against original or previous strains. An example of such disease is an emerging virus called the corona virus 2019 (COVID-19) virus that has infected and killed millions around the world within a period of two years. The virus was caused by the virus species ‘severe acute respiratory syndrome corona virus’, named SARS-CoV-2. The airborne transmission occurs by inhaling droplets loaded with SARS-CoV-2 particles that are expelled by infectious people. Symptoms of the virus appear 2–14 days of exposure to certain strains of the virus. Several SARS-CoV-2 variants have emerged around the world, with each new variants having different characteristics. The COVID-19 virus evolves as changes in its genetic code occur during replication of the genome. The United States Centers for Disease Control and Prevention (CDC) and the US government SARS-COV-2 Inter-agency Group (SIG) [1] have confirmed the emergence of at least twelve new variants of the virus. The SIG group evaluates the risk of the circulating SARS-CoV-2 variants in the United States and make recommendations about the impact, severity, and how spread the virus is. Lineages of these variants that are classified as variant being monitored (VBM) and designated as Variant of Concerned (VOC) in the United States may lead to more severe cases of the virus. By lineages, we mean a group of related virus variant from a common ancestor. The variant, called Omicron-B.1.1.529 [2] was first detected in specimens collected on November 11, 2021 in Botswana and on November 14, 2021 in South Africa. It was first detected in the United States on December 1, 2021 and classified as a VOC. There are increased attention given to the Omicron variant. It is now the main circulating variant in the United States as of early January, 2022, accounting for about 95% of the US reported cases because of its high infection rate. Studies show that even vaccinated individuals can still be infected with this new variant if proper care is not taken. Scientist around the world are working to learn more about this new variant, and to gather more data for the purpose of studying if the variant causes more illness, hospitalizations, and death than infection with other variants. As of early December 2021, one of the new variants, called the Delta-B.1.617.2 variant was said to be the main circulating variant in the United States, and also classified as variant of concern by the SIG group. It was first identified in India and was reported to spread much faster and causes more severe cases than other early variants in the United States, probably causing twice [3] as many infections. Other variants, called the Alpha-B.1.1.7, Beta-B.1.351, Gamma-P.1, Epsilon B.1.427 & B.1.429, Eta B.1.525, Iota B.1.526, Kappa B.1.617.1, Zeta P.2, Mu B.1.621, are classified as VBM. The Alpha-B.1.1.7, Beta-B.1.351, and Gamma-P.1 variants were first discovered in the United Kingdom, South Africa, and Japan/Brazil, respectively. We aim to study how these variants are being transmitted and the impact that vaccines are having on mitigating the number of infection cases in a particular population.

Several mathematical models [4–36] have been developed to study the transmission of infectious diseases. Some of these works [5, 11, 20, 24, 31, 32, 37–43] discussed the transmission of infectious disease caused by the variants and lineages of the COVID-19 virus. SIR models including a modified SIR model with two strains and vaccinated group [10], a generalized SIR model with *n*- strains [39], a SIR model with complete cross-protection and nonlinear force of infection [44], a coupled multi-strain SIR epidemic model [24] have been developed to describe the transmission of the COVID-19 strains. Other works such as a multi-strain SEIR models with optimal control [5, 6], multi-strain SEIR models with saturated and general incidence rates [5, 15], SIRD [45] and SEIPAHRF model [46] with Caputo fractional derivative, SCIRP model incorporating media influence [47], and statistical analysis [48] have also been considered in describing the transmission of the virus and its strains. We direct the readers to the work of Hattaf et al. [49, 50] for more recent information about the fractional differential equations and its generalization.

Viruses undergo changes and these changes are cause for concern for people who have recovered from the virus, and also to those who are vaccinated against certain strain of the virus. As discussed in Fudolig et al. [10], a highly infectious emergent strain can infect the susceptible population before the original strain, thereby impeding the spread of the original strain or causing the two strains to coexist in an endemic equilibrium. For this reason, the need to determine conditions in which a newly emerged strain and an existing strains that have a means of immunity will coexist in a population is of utmost important.

Most of the papers mentioned above often utilize simpler versions of the multi-strain network, and provides less mathematical details on the asymptotic behavior of the model. In this work, we develop a multi-strain compartmental model by assuming a population is completely susceptible to *n*- different variants of a particular virus at the beginning of an epidemic, with the population of the region partitioned into compartments consisting of susceptible population, population vaccinated against strain *k* (1 ≤ *k* ≤ *n*) of the virus, population exposed to strain *k* of the virus, infected asymptomatic population with strain *k* of the virus, infected symptomatic population with strain *k* of the virus, and population that recovered from strain *k* of the infection. By denoting *P* = {1, 2, ⋯, *n*} and $S_{r}\in 2^{P}$ as a subset of the power set 2^*P*^ with *r* number of strains, *r* = 0, 1, ⋯, *n*, with *r* = 0 representing disease-free case, we study the existence and stability conditions for the equilibrium point corresponding to the scenario where only strains in $S_{r}$ survive. This result is used to estimate the secondary number of infections produced by strain-*k* infected individual when introduced into a completely susceptible population. This number helps public health expert control the spread of the virus as new variants emerge. Conditions under which an endemic with more than one strain of the virus exist are calculated. The question as to whether this is possible is answered by studying the stability analysis of endemic strains. This model also helps us to understand the correlation between the daily number of administered vaccines and the number of infected, exposed, and recovered population in the region better. To understand the disease dynamics better, we study a powerful quantitative concept that can be used to characterize the contagiousness of each strain of the infectious disease and how transmissible they are. The basic reproduction number, which is the expected number of secondary cases produced by a typical infectious individual in a completely susceptible population in the presence and absence of vaccination are calculated. This study also helps to shed more light on the possibility of a disease being eliminated from a population if enough individuals are immune due to either vaccination or recovery from prior exposure to the disease. A bound for the herd immunity threshold, which is the minimum proportion of the population that must be vaccinated in order to stop the spreading of the disease in the population is calculated and analyzed for each variants.

This paper is organized as follows: In Section 2, a model is developed for the transmission of multi strain infectious diseases for the case where individuals vaccinated against specific strains are immune to that strain and its predecessors but can still be infected by newer emerging strains.The validity of the model, together with the existence and uniqueness of its solution is proved in this section. The reproduction numbers for the cases where the population is vaccinated and when it is not vaccinated are calculated. With these, the effect of vaccination in mitigating infection is studied. Existence of equilibrium points for the case where certain strains persist in the population is examined. In Section 3, the local and global stability of all equilibrium points for model (1) is investigated. In addition to the assumption made in Section 2, an extension of model (1) for the case where those that recovered from a particular strain can get infected by emerging strain is derived and analyzed in Section 4. Existence and stability of equilibrium points for the model is also discussed. Numerical simulations are carried out in Section 5 by applying models (1) and (35) to analyze COVID-19 data. Summary of the work done in this work is discussed in Section 6.

## 2 Materials and methods

### 2.1 Model formulation

By assuming a population is completely susceptible to *n*- different variants of a particular virus at the beginning of an epidemic, we partitioned the population of the region into compartments consisting of susceptible (denoted *S*(*t*)) population, population *V*_*k*_(*t*) vaccinated against strain *k* of the virus, population *E*_*k*_(*t*) of exposed individuals infected with strain/variant *k* of the virus, infected asymptomatic population *A*_*k*_(*t*) with strain/variant *k* of the virus, infected symptomatic population *I*_*k*_(*t*) with strain/variant *k* of the virus, and population *R*_*k*_(*t*) that recovered from strain *k* of the virus at a given time *t*, for *k* = 1, 2, ⋯, *n*. These state variables are described in Table 1. We assume that the order of existence of newer strains follow *k* = 1, 2, ⋯, *n*. That is, the population is first infected by strain 1, followed by strain 2, ⋯, *n*. We assume those who are vaccinated and recovered from strain *k* are immune to previous and present strains *j* = 1, 2, ⋯, *k*, but can be infected by newer strains *j* = *k* + 1, *k* + 2, ⋯, *n*. We can see, for example, that this assumption is satisfied in the case of COVID-19 and supported by the United States Centers for Disease Control and Prevention (CDC), who claimed that current vaccines approved in the United States are expected to protect against severe illness, hospitalizations, and deaths caused by variants of the virus, although people who are fully vaccinated can still be infected. We assume that the migration rate into the susceptible population not receiving vaccination against any of the strains of the virus is (1 − *q*)*μ*, while the migration rate into the population vaccinated against strain *k* is *μq*_*k*_, where $q=\sum_{k=1}^{n}q_{k}<1$. The parameter *β*_*j*_ denotes the per capita contact rate of susceptible individuals with symptomatic infected strain *j*; *γ*_*j*_ denotes the per capita contact rate of susceptible individuals with asymptomatic infected strain *j*; ${\bar{\beta }}_{j,k}$ and ${\bar{\gamma }}_{j,k}$ denote the reduced per capita contact rates of symptomatic and asymptomatic strain *j* infected individual, respectively, that are vaccinated against strain *k*, *j* = *k* + 1, ⋯, *n*; *h*_*j*, *k*_ and *ϵ*_*j*, *k*_ denote the per capita contact rates of symptomatic and asymptomatic strain *j* infected individual, respectively, that recovered from strain *k*, *j* = *k* + 1, ⋯, *n*. These parameters are described in detail in Table 2.

**Table 1:** Description of variables for the epidemic model.

Variable	Description
*S*	Population of susceptible individuals
*V*_*k*_	Population of vaccinated individuals immune to strain *k* of the infection
*E*_*k*_	Population of individuals exposed to strain *k* of the infection
*A*_*k*_	Population of asymptomatic individuals infected with strain *k* of the infection
*I*_*k*_	Population of symptomatic individuals infected with strain *k* of the infection
*R*_*k*_	Population of individuals who recovered from the *k*-th strain

**Table 2:** Description of parameters for the epidemic model.

Parameter	Description
*β*_*k*_	Transmission rate of symptomatic infected individuals with strain *k* of infection interacting with susceptible population
*γ*_*k*_	Transmission rate of asymptomatic infected individuals with strain *k* of infection interacting with susceptible population
${\bar{\beta }}_{k,j}$	Transmission rate of symptomatic infected individuals with strain *k* interacting with population vaccinated against strain *j* of infection, *k* = *j* + 1, ⋯, *n*
${\bar{\gamma }}_{k,j}$	Transmission rate of asymptomatic infected individuals with strain *k* interacting with population vaccinated against strain *j* of infection, *k* = *j* + 1, ⋯, *n*
*h*_*k*, *j*_	Transmission rate of symptomatic infected individuals with strain *k* interacting with population that recovered from strain *j* of infection, *k* = *j* + 1, ⋯, *n*
*ϵ*_*k*, *j*_	Transmission rate of asymptomatic infected individuals with strain *k* interacting with population that recovered from strain *j* of infection, *k* = *j* + 1, ⋯, *n*
*μ*	Natural birth rate
*p*	Fraction of infection cases that are asymptomatic
*q*_*k*_	Fraction of population vaccinated against strain *k* of infection
λ_*k*_	Transition rate of individuals with strain *k* of infection from exposed to infected class
*r*_*k*_	Asymptomatic recovery rate of those with strain *k* of infection
*θ*_*k*_	Symptomatic recovery rate of those with strain *k* of infection
*q*	$\sum_{j=1}^{n}q_{j}$

In this section, we study a case where individuals vaccinated against specific strains are immune to that strain and its predecessors but can still be infected by newer emerging strains. Without loss of generality, we assume the population is normalized so that the sizes *S*, *V*_*k*_, *E*_*k*_, *A*_*k*_, *I*_*k*_, and *R*_*k*_ are in percentages, for *k* = 1, 2, ⋯, *n*. The model governing the transmission of the infectious disease for this case follows the system of deterministic differential equation

$$\begin{array}{c} \begin{array}{ccc} dS & = & (\left(1-q\right)\mu -S\sum_{j=1}^{n}(\beta_{j}I_{j}+\gamma_{j}A_{j})-\mu S)dt,\;\;S\left(t_{0}\right)=S_{0},\\ dV_{k} & = & (q_{k}\mu -V_{k}\sum_{j=k+1}^{n}({\bar{\beta }}_{jk}I_{j}+{\bar{\gamma }}_{jk}A_{j})-\mu V_{k})dt,\;\;V_{k}\left(t_{0}\right)=V_{k0},\;\;k=1,2,\cdots ,n-1\\ dV_{n} & = & (q_{n}\mu -\mu V_{n})dt,\;\;V_{n}\left(t_{0}\right)=V_{n0},\\ dE_{1} & = & (S(\beta_{1}I_{1}+\gamma_{1}A_{1})-e_{1}E_{1})\;dt,\;\;E_{1}\left(t_{0}\right)=E_{10},\\ dE_{k} & = & (S(\beta_{k}I_{k}+\gamma_{k}A_{k})+\sum_{j=1}^{k-1}V_{j}({\bar{\beta }}_{kj}I_{k}+{\bar{\gamma }}_{kj}A_{k})-e_{k}E_{k})\;dt,\;E_{k}\left(t_{0}\right)=E_{k0},\;k=2,3,\cdots ,n\\ dA_{k} & = & (p\lambda_{k}E_{k}-a_{k}A_{k})\;dt,\;\;k=1,2,\cdots ,n\;\;\;A_{k}\left(t_{0}\right)=A_{k0},\;\;k=1,2,\cdots ,n\\ dI_{k} & = & (\left(1-p\right)\lambda_{k}E_{k}-w_{k}I_{k})\;dt,\;\;I_{k}\left(t_{0}\right)=I_{k0},\;\;k=1,2,\cdots ,n,\\ dR_{k} & = & (\theta_{k}I_{k}+r_{k}A_{k}-\mu R_{k})dt,\;\;R_{k}\left(t_{0}\right)=R_{k0},\;\;k=1,2,\cdots ,n-1\\ dR_{n} & = & (\theta_{n}I_{n}+r_{n}A_{n}-\mu R_{n})\;dt,R_{n}\left(t_{0}\right)=R_{n0}, \end{array} \end{array}$$ (1)

where

$$\begin{array}{c} \begin{array}{ccc} e_{k} & = & \mu +\lambda_{k},\\ a_{k} & = & \mu +r_{k},\\ w_{k} & = & \mu +\theta_{k}, \end{array} \end{array}$$ (2)

and the states and parameters in the model are described in Tables 1 and 2, respectively.. An extension of model (1) to include a case where those that recovered from a particular strain can get infected by emerging strain is discussed in Section 4.

**Fig 1:**
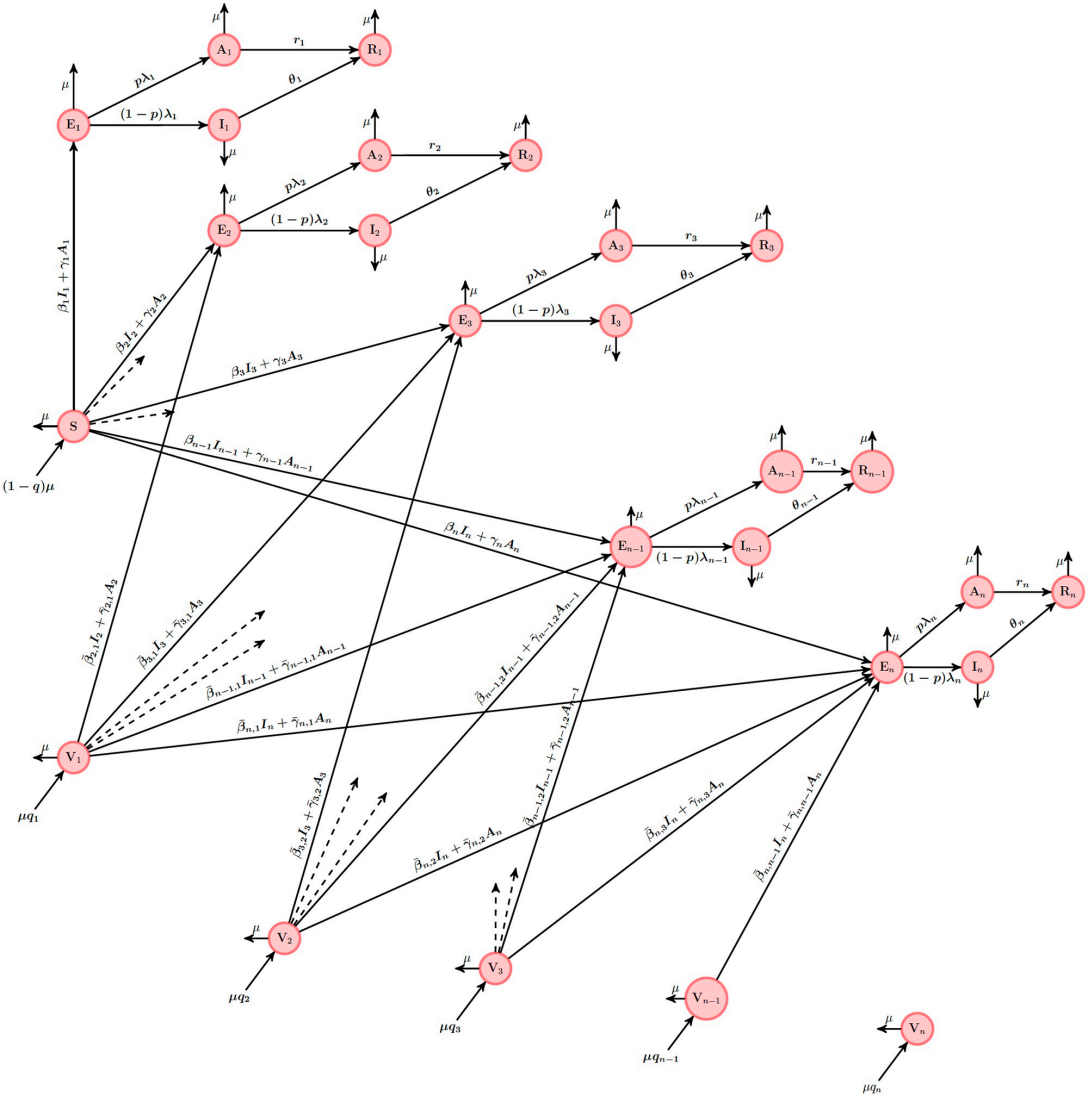
Schematic diagram for the epidemic model (1). The circle compartments represent group of individuals.

In order to better understand the transmission dynamics described in (1), we give a schematic diagram of the model in Fig 1. The circle compartments represent group of individuals. An arrow pointing out of a compartment represents migration of individuals out of the compartment.

**Remark 1.** *Since individuals in the vaccinated group V*_*j*_ *are only assumed to be immune to strain j and its predecessors, and not to future strains k* > *j*, *we assume they are also susceptible to future strains k* > *j*. *For this reason, we expect that the rate at which an infectious individual with strain k make contact with susceptible individuals and individuals in group V*_*j*_, *j* < *k*, *should be the same. That is*, $\beta_{k}={\bar{\beta }}_{k,j}$ *for j* = 1, 2, ⋯, *k* − 1. *Likewise, we expect β*_*k*_ = *h*_*k*, *j*_, $\gamma_{k}={\bar{\gamma }}_{k,j}=\in_{k,j}$, *for j* = 1, 2, ⋯, *k* − 1. *Vaccination against past and current strains do not provide any protection against new emerging strains. In the case where the infectivity of strain k* (*k* > *j*) *is different for the vaccinated group V*_*j*_ *due to certain circumstances so that* $\beta_{k}\neq {\bar{\beta }}_{k,j}$ *and* $\gamma_{k}\neq {\bar{\gamma }}_{k,j}$, *we present a comparison of the reproduction number for the case where* $\beta_{k}={\bar{\beta }}_{k,j}$, $\gamma_{k}={\bar{\gamma }}_{k,j}$ and the case where $\beta_{k}>{\bar{\beta }}_{k,j}$, $\gamma_{k}>{\bar{\gamma }}_{k,j}$ for *j* = 1, 2, ⋯, *k* − 1 *in Remark 3. Also, based on model* (1), *an individual infected with certain strain k cannot be infected with another strain j* ≠ *k at a given time t*.

### 2.2 Validity of the epidemic model (1)

In this section, we discuss the validity of the proposed epidemic model (1), that is, we discuss the existence and uniqueness of the solution of (1). Let $N=S+\sum_{k=1}^{n}(V_{k}+E_{k}+A_{k}+I_{k}+R_{k})$. Since the population is assumed to be normalized, we have $N_{0}=N\left(t_{0}\right)=S_{0}+\sum_{k=1}^{n}(V_{k0}+E_{k0}+A_{k0}+I_{k0}+R_{k0})=1$ denoting the entire population. It follows that *dN*/*dt* = *μ* − *μN*, *N*(*t*_0_) = 1, so that the population size is constant over time. This suggests that the birth and death rates (with the recruitment rate into the susceptible *S* and vaccinated *V*_*k*_ classes in the absence and presence of vaccines being (1 − *q*)*μ* and *q*_*k*_*μ*, respectively, *k* = 1, 2, ⋯, *n*) are assumed to be the same so that the population is constant over certain period of time. The most appropriate epidemiological feasible region where solution exist is the set

$$\begin{array}{c} T=\{(S,{\left\{V_{k}\right\}}_{k=1}^{n},{\left\{E_{k}\right\}}_{k=1}^{n},{\left\{A_{k}\right\}}_{k=1}^{n},{\left\{I_{k}\right\}}_{k=1}^{n},{\left\{R_{k}\right\}}_{k=1}^{n})\in R_{+}^{5n+1}\;|\;0\leq S+\sum_{k=1}^{n}(V_{k}+E_{k}+A_{k}+I_{k}+R_{k})\leq 1\}. \end{array}$$ (3)

The existence, uniqueness, and positiveness of the solution of (1) is shown for the case where *S*_0_ > 0, *V*_*k*0_ > 0, *E*_*k*0_ > 0, *A*_*k*0_ > 0, *I*_*k*0_ > 0, *R*_*k*0_ > 0 using results from Kelley and Peterson [51].

**Theorem 1.** *If S*_0_ > 0, *V*_*k*0_ > 0, *E*_*k*0_ > 0, *A*_*k*0_ > 0, *I*_*k*0_ > 0, *R*_*k*0_ > 0, *k* = 1, 2, ⋯, *n, then there exist a positive unique solution of* (1) *in the feasible region* T *for all t* ≥ 0.

*Proof.* Let *S*_0_ > 0, *V*_*k*0_ > 0, *E*_*k*0_ > 0, *A*_*k*0_ > 0, *I*_*k*0_ > 0, *R*_*k*0_ > 0 for model (1). Define

$$\begin{array}{c} \tilde{y}={\left(S,V_{1},\cdots ,V_{n},E_{1},\cdots ,E_{n},A_{1},\cdots ,A_{n},I_{1},\cdots ,I_{n},R_{1},\cdots ,R_{n}\right)}^{T} \end{array}$$

so that Eq (1) can be written as

$$\begin{array}{c} d\tilde{y}=h\left(t\right)\;dt,\;\;\tilde{y}\left(t_{0}\right)={\tilde{y}}_{0}, \end{array}$$ (4)

for some vector function *h*(*t*). It can be shown that $h\;:\;R^{5n+1}\to R^{5n+1}$ is continuous with continuous first-order partial derivatives with respect to ${\tilde{y}}_{1},{\tilde{y}}_{2},\cdots ,{\tilde{y}}_{5n+1}$. It follows from Theorem 3.1 of Kelley and Peterson [51] that there exist a unique solution *S*(*t*), *V*_*k*_(*t*), *E*_*k*_(*t*), *A*_*k*_(*t*), *I*_*k*_(*t*), *R*_*k*_(*t*), *k* = 1, 2, ⋯, *n* of (1) for all *t* ≥ 0. Let $\lambda \left(t\right)=\sum_{j=1}^{n}(\beta_{j}I_{j}\left(t\right)+\gamma_{j}A_{j}\left(t\right))$. It follows from (1) that *S*(*t*) satisfies $S\left(t\right)=S_{0}\;\text{exp}(-\int_{0}^{t}(\mu +\lambda (v))dv)+\left(1-q\right)\mu \;\text{exp}(-\int_{0}^{t}(\mu +\lambda (v))dv)\int_{0}^{t}\text{exp}(-\int_{0}^{u}(\mu +\lambda (v))dv)du>0$ for all *t* ≥ 0. Likewise, we can show in a similar manner that *V*_*k*_(*t*) > 0, *E*_*k*_ > 0, *A*_*k*_(*t*) > 0, *I*_*k*_(*t*) > 0, and *R*_*k*_(*t*) > 0 for all *k* = 1, 2, ⋯, *n*, *t* ≥ 0. The result follows since *dN*/*dt* = *μ* − *μN*, *N*(*t*_0_) = 1.

The long term behavior of the solutions of model (1) depends on certain thresholds of the reproduction number. The reproduction number and the thresholds are calculated in sections to come.

### 2.3 Reproduction number

Define

$$\begin{array}{c} \begin{array}{ccc} c_{k} & = & \left(1-p\right)a_{k}\beta_{k}+pw_{k}\gamma_{k},\\ {\bar{c}}_{k} & = & \left(1-p\right)a_{k}\theta_{k}+pw_{k}r_{k},\;\;\text{for}\;k=1,2,\cdots ,n. \end{array} \end{array}$$ (5)

The disease-free equilibrium, denoted $E_{0}$, of the system (1) is given by

$$\begin{array}{c} E_{0}=\left\{S^{0}=1-q,V_{1}^{0}=q_{k},\cdots ,V_{n}^{0}=q_{n},E_{1}^{0}=0,\cdots ,E_{n}^{0}=0,A_{1}^{0}=0,\cdots ,A_{n}^{0}=0,I_{1}^{0}=0,\cdots ,I_{n}^{0}=0,R_{1}^{0}=0,\cdots ,R_{n}^{0}=0\right\}. \end{array}$$ (6)

The dynamic model (1) can be written in the form

$$\begin{array}{c} dx=(F(x)-V(x))\;dt, \end{array}$$ (7)

where

$$\begin{array}{c} x={\left(E_{1},\cdots ,E_{n},A_{1},\cdots ,A_{n},I_{1},\cdots ,I_{n},R_{1},\cdots ,R_{n},V_{1},\cdots ,V_{n},S\right)}^{T}, \end{array}$$ (8)

$F_{j}$ denotes the rate of appearance of new infections in compartment *j*, $V_{j}=V_{j}^{-}-V_{j}^{+}$ with $V_{j}^{+}$ and $V_{j}^{-}$ denoting the rate of transfer of individuals in and out of compartment *j*, respectively [52]. For any vector **u** = (*u*_1_⋯, *u*_*n*_)^*T*^, define the *n* × *n* matrix ***M***(**u**) by the diagonal matrix

$$\begin{array}{c} M(u)=\text{diag}(u_{1},u_{2},\cdots ,u_{n}). \end{array}$$ (9)

Define

$$\begin{array}{c} Q_{k}=\sum_{j=1}^{k}q_{j-1}, \end{array}$$ (10)

where *q*_0_ = 0. Let $\tilde{\beta }$ and $\tilde{\gamma }$ be two vectors with entries

$$\begin{array}{c} {\tilde{\beta }}_{k}=(1-q+Q_{k})\beta_{k},\\ {\tilde{\gamma }}_{k}=(1-q+Q_{k})\gamma_{k}, \end{array}$$

respectively, where ${\tilde{\beta }}_{1}=\left(1-q\right)\beta_{1}$ and ${\tilde{\gamma }}_{1}=\left(1-q\right)\gamma_{1}$. Define **F** and **V** such that

$$\begin{array}{c} (F_{i,j})=(\frac{\partial F_{i}}{\partial x_{j}}\left(E_{0}\right)),\\ (V_{i,j})=(\frac{\partial V_{i}}{\partial x_{j}}\left(E_{0}\right)), \end{array}$$

where (*i*, *j*) with 1 ≤ *i*, *j* ≤ 3*n* corresponds to the index of the infected compartments. Based on our model (1), there are three infected compartments *E*, *A*, and *I*, each assumed to have *n*-different strains so that the infected compartments are the first 3*n* entries of **x** in (8). Let **0**_*n* × *n*_ represents the zero square matrix of order *n*. The corresponding matrices *F* and *V* are calculated as

$$\begin{array}{ccc} F & = & (\begin{array}{ccc} 0_{n\times n} & M(\tilde{\gamma }) & M(\tilde{\beta })\\ 0_{n\times n} & 0_{n\times n} & 0_{n\times n}\\ 0_{n\times n} & 0_{n\times n} & 0_{n\times n} \end{array}),\\ V & = & (\begin{array}{ccc} M(e) & 0_{n\times n} & 0_{n\times n}\\ -pM(\lambda ) & M(a) & 0_{n\times n}\\ -\left(1-p\right)M(\lambda ) & 0_{n\times n} & M(w) \end{array}), \end{array}$$

where ***M***(**u**) is as defined in (9) and $\tilde{\beta }$, $\tilde{\gamma }$, ***e***, ***λ***, ***a***, and ***w*** are vectors. From the matrix

$$\begin{array}{c} V^{-1}=(\begin{array}{ccc} M{\left(e\right)}^{-1} & 0_{n\times n} & 0_{n\times n}\\ pM{\left(e\right)}^{-1}M{\left(a\right)}^{-1}M(\lambda ) & M{\left(a\right)}^{-1} & 0_{n\times n}\\ \left(1-p\right)M{\left(e\right)}^{-1}M{\left(w\right)}^{-1}M(\lambda ) & 0_{n\times n} & M{\left(w\right)}^{-1} \end{array}), \end{array}$$

where ***M***(***e***)^−1^ is the diagonal matrix $M{\left(e\right)}^{-1}=\text{diag}(\frac{1}{e_{1}},\frac{1}{e_{2}},\cdots ,\frac{1}{e_{n}})$, we see that the average length of time an individual spent being exposed, asymptomatically infected, symptomatically infected with strain *k* is 1/*e*_*k*_, 1/*a*_*k*_, and 1/*w*_*k*_, respectively. Also, the average length of time an individual exposed to strain *k* spent being asymptomatic with the same strain during its life time is expected to be $\frac{pw_{k}\lambda_{k}}{a_{k}e_{k}w_{k}}$. The average length of time an individual exposed to strain *k* spent being symptomatic with strain *k* during its life time is expected to be $\frac{\left(1-p\right)a_{k}\lambda_{k}}{a_{k}e_{k}w_{k}}$. Since the transmission rates of symptomatic and asymptomatic individuals with strain *k* in a susceptible population are *β*_*k*_ and *γ*_*k*_, respectively, it follows that at the emergence of a new strain *k* (*k* ≥ 2), the expected number of new infections produced by individual with such strain in a completely susceptible non-vaccinated population, and a population completely vaccinated against strain *j*, are $\frac{\left(1-q\right)(\left(1-p\right)a_{k}\beta_{k}+pw_{k}\gamma_{k})\lambda_{k}}{a_{k}e_{k}w_{k}}=\frac{\left(1-q\right)c_{k}\lambda_{k}}{a_{k}e_{k}w_{k}}$ and $\frac{q_{j-1}(\left(1-p\right)a_{k}\beta_{k}+pw_{k}\gamma_{k})\lambda_{k}}{a_{k}e_{k}w_{k}}=\frac{q_{j-1}c_{k}\lambda_{k}}{a_{k}e_{k}w_{k}}$, respectively, *j* = 1, 2, ⋯, *n*, where we set *q*_0_ = 0.

The expected number, $R_{k}$, of new infections produced by individual with such strain *k* in a population containing susceptible and vaccinated individuals is obtained as

$$\begin{array}{c} R_{k}=\frac{\left(1-q\right)c_{k}\lambda_{k}+\sum_{j=1}^{k}q_{j-1}c_{k}\lambda_{k}}{a_{k}e_{k}w_{k}}=\frac{(1-q+Q_{k})c_{k}\lambda_{k}}{a_{k}e_{k}w_{k}},\;\;k=1,2,\cdots ,n, \end{array}$$ (11)

where 1 − *q* is the proportion of those that are susceptible but not vaccinated. Using the next generation matrix [52], since **F** is non-negative and **V** is a non-singular *M*-matrix [53], the number $R_{k}$ obtained in (11) is the (*k*, *k*)-th entry of the next generation matrix **FV**^−1^, for *k* = 1, 2, ⋯, *n*. It is the expected number of new infections in compartment *k* (compartment exposed to strain *k*) produced by infected individual originally introduced into the same compartment.

**Remark 2.** *Effect of Vaccination*

*We remark here that the expected number* $R_{k}$ *of new infections in the compartment exposed to strain k depends on the vaccination rates q*_*l*_, *l* = *k*, *k* + 1, ⋯, *n*. *If no one is vaccinated in the population (that is, if q*_*l*_ = 0 *for all l* = 1, 2, ⋯, *n)*, *then the expected number of infections caused by strain k is obtained to be*

$$\begin{array}{c} R_{c,k}=\frac{c_{k}\lambda_{k}}{a_{k}e_{k}w_{k}},\;\;k=1,2,\cdots ,n. \end{array}$$ (12)

*It follows from Remark 1 that this number is clearly more than the reproduction number obtained in* (11) *where some individuals are receiving vaccination in the population. The reproduction number* $R_{k}$ *can be written in terms* $R_{c,k}$ *as*

$$\begin{array}{c} R_{k}=(1-q+Q_{k})R_{c,k}, \end{array}$$ (13)

*showing that the ratio of numbers of infection caused by strain k in a population with vaccination to a population without vaccination is* 1 − *q* + *Q_k_* : 1. *If individuals are not vaccinated against strain k (that is, if q_k_* = 0 *for fixed k), then the expected number of infections caused by strain k increases to*

$$\frac{\left(1-\sum_{\begin{array}{l} j=1\\ j\neq k \end{array}}^{n}q_{j}\right)c_{k}\lambda_{k}+\sum_{j=1}^{k}q_{j-1}c_{k}\lambda_{k}}{a_{k}e_{k}w_{k}}=\frac{\left(1-\sum_{j=k+1}^{n}q_{j}\right)c_{k}\lambda_{k}}{a_{k}e_{k}w_{k}},$$

*which is the same as* $R_{k}+\frac{q_{k}c_{k}\lambda_{k}}{a_{k}e_{k}w_{k}}$. *This shows that a non vaccinated strain k infected individual produces* $\frac{q_{k}c_{k}}{\left(1-q\right)c_{k}+\sum_{j=1}^{k-1}q_{j}c_{k}}\times 100\%$ *more infections than a vaccinated strain k infected individual. If on the other hand, everyone in the population is vaccinated (that is, q* = 1*), then the expected number of infections caused by strain k significantly reduced to*

$$\begin{array}{c} \frac{\sum_{j=1}^{k}q_{j-1}c_{k}\lambda_{k}}{a_{k}e_{k}w_{k}},\;\;k=1,2,\cdots ,n. \end{array}$$

*That is, a completely vaccinated population produces* $\frac{1-q}{1-q+Q_{k}}\times 100\%$ *lesser infections than population not completely vaccinated. These analyses show the importance of being vaccinated in the population*.

The reproduction number $R_{0}$ for the system, which is defined as the expected number of secondary infections produced by a typical infected individuals over the course of its infectious period, is calculated using the next generation matrix approach by van den Diessche et al. [52] as

$$\begin{array}{c} R_{0}=\max_{1\leq k\leq n}\{(1-q+Q_{k})\frac{c_{k}\lambda_{k}}{a_{k}e_{k}w_{k}}\}. \end{array}$$ (14)

We shall later show that if $R_{0}<1$, then we expect a typical infected individual to produce less than one new infected individual, meaning all strains of the disease will eventually die out in the population (in the presence of vaccination). Although it is well known that a value of the reproduction number greater than one means that epidemic will persist in the population [8, 52], we shall later show that a strain with a reproduction number greater than 1 can still die out on the long run if a newer emerging strain has a greater reproduction number. The value $R_{k}$ on the other hand, can be interpreted as the reproduction number for typical individual infected with strain *k* of the disease, for *k* = 1, 2, ⋯, *n*.

**Remark 3.** *In the case where* $\beta_{k}\neq {\bar{\beta }}_{k,j}$ *and* $\gamma_{k}\neq {\bar{\gamma }}_{k,j}$ *such that* $\beta_{k}>{\bar{\beta }}_{k,j}$ *and* $\gamma_{k}>{\bar{\gamma }}_{k,j}$ *for all* 1 ≤ *k*, *j* ≤ *n due to some form of partial immunity as a result of vaccines, then the strain k reproduction number* $R_{k}$ *and the reproduction number* $R_{0}$ *in* (11) *and* (14)*, respectively, are obtained as*

$$\begin{array}{c} \begin{array}{c} R_{k}=\frac{\left(1-q\right)c_{k}\lambda_{k}+\sum_{j=1}^{k}q_{j-1}{\hat{c}}_{k,j-1}\lambda_{k}}{a_{k}e_{k}w_{k}},\;\;k=1,2,\cdots ,n,\\ R_{0}=\max_{1\leq k\leq n}\{\frac{\left(1-q\right)c_{k}\lambda_{k}+\sum_{j=1}^{k}q_{j-1}{\hat{c}}_{k,j-1}\lambda_{k}}{a_{k}e_{k}w_{k}}\}, \end{array} \end{array}$$ (15)

where

$$\begin{array}{c} {\hat{c}}_{k,j}=\left(1-p\right)a_{k}{\bar{\beta }}_{k,j}+pw_{k}{\bar{\gamma }}_{k,j},\;\;j=1,\cdots ,k-1, \end{array}$$ (16)

*and we set* ${\hat{c}}_{k,0}=0$ ∀ *k* = 1, 2, ⋯, *n*. *It is easy to show that* $c_{k}>{\hat{c}}_{k,j}$ *in this case, so that the estimates of the reproduction numbers given in* (15) *are greater than those given in* (11) *and* (14)*, respectively. This shows that the reproduction number reduces as vaccines reduce the infectivity of the viruses (as expected)*.

**Remark 4.** *The reproduction number* $R_{0}$ *is for the case where some members of the population are vaccinated against certain strain of the disease, that is, case with compartments V*_1_, *V*_2_, ⋯, *V*_*n*_ *in the population. As explained in Remark 2, the reproduction number, denoted* $R_{c}$, *for a completely susceptible non-vaccinated population is given by*

$$\begin{array}{c} R_{c}=\max_{1\leq k\leq n}\{\frac{c_{k}\lambda_{k}}{a_{k}e_{k}w_{k}}\}. \end{array}$$ (17)

### 2.4 Existence of equilibrium points

We discuss conditions under which equilibrium points of (1) exist. System (1) has many equilibrium points. Let *P* = {1, 2, ⋯, *n*} denotes set of indices representing order of existence of new strain in the population, with 2^*P*^ denoting the power set of *P*. Let $S_{r}\in 2^{P}$ denotes a subset of 2^*P*^ with *r* number of strains, *r* = 0, 1, ⋯, *n*, with *r* = 0 representing disease-free case. We study the existence conditions for the equilibrium point corresponding to the scenario where only strains in $S_{r}$ survives, *r* = 0, 1, ⋯, *n*. The case *r* = 0 exists for the disease-free equilibrium $E_{0}$ case. The disease-free equilibrium is given in (6). For the case *r* = 1 representing case where only one strain survives, we shall denote the strain by strain *m*, *m* = 1, 2, ⋯, *n*, and the equilibrium referred to as the strain *m* equilibrium point and denoted $E_{m}$. We study the conditions under which such equilibrium point exists, and in general, we also study conditions under which strains with indices in $S_{r}$ survives, for *r* = 1, 2, ⋯, *n*.

**Theorem 2.** *The strain m unique equilibrium point, denoted* $E_{m}$, *for the epidemic model* (1) *exists in the feasible region* T *provided* $R_{m}>1$.

*Proof.* The strain *m* equilibrium point $E_{m}$ is obtained by solving the equation *h*(*t*) = 0 and setting *E*_*k*_ = *A*_*k*_ = *I*_*k*_ = *R*_*k*_ = 0 for 1 ≤ *k* ≠ *m* ≤ *n*, where *h*(*t*) is a vector in (4) containing the right hand side of (1). It follows from the equation governing *A*_*m*_ and *I*_*m*_ that $A_{m}=\frac{p\lambda_{m}}{a_{m}}E_{m}$ and $I_{m}=\frac{\left(1-p\right)\lambda_{m}}{w_{m}}E_{m}$. Substituting these into the equations governing *S*, *V*_*k*_, *k* = 1, 2, ⋯, *n*, *E*_*m*_, and *R*_*m*_, we have

$$\begin{array}{ccc} S & = & \frac{(1-q)\mu }{\mu +\frac{c_{m}\lambda_{m}}{a_{m}w_{m}}E_{m}},\\ V_{k} & = & \left\{ \begin{array}{c} \frac{q_{k}\mu }{\mu +\frac{c_{m}\lambda_{m}}{a_{m}w_{m}}E_{m}},\;\;\text{if}\;\;k<m,\\ q_{k},\;\;\text{if}\;\;k\geq m, \end{array}\right.\\ E_{m} & = & \frac{1}{c_{m}\lambda_{m}e_{m}}(\mu c_{m}\lambda_{m}\left(1-q\right)+\mu c_{m}\lambda_{m}Q_{m}-\mu a_{m}w_{m}e_{m}),\\ R_{m} & = & \frac{\left(1-p\right)a_{m}\theta_{m}+pr_{m}w_{m}}{\mu a_{m}w_{m}}\lambda_{m}E_{m}, \end{array}$$

where *c*_*m*_ and ${\bar{c}}_{m}$ are defined in (5). If $R_{m}>1$, the strain *m* equilibrium point $E_{m}$ is obtained as

$$\begin{array}{c} E_{m}=\left\{S^{*},V_{1}^{*},\cdots ,V_{n}^{*},E_{1}^{*},\cdots ,E_{n}^{*},A_{1}^{*},\cdots ,A_{n}^{*},I_{1}^{*},\cdots ,I_{n}^{*},R_{1}^{*},\cdots ,R_{n}^{*}\right\}, \end{array}$$ (18)

where

$$\begin{array}{c} \begin{array}{ccc} E_{m}^{*} & = & \frac{\mu a_{m}w_{m}}{c_{m}\lambda_{m}}(R_{m}-1),\\ S^{*} & = & \frac{\left(1-q\right)}{R_{m}}\\ V_{k}^{*} & = & \left\{ \begin{array}{c} \frac{q_{k}}{R_{m}},\;\;\text{if}\;\;k<m,\\ q_{k},\;\;\text{if}\;\;k\geq m, \end{array}\right.\\ A_{m}^{*} & = & \frac{p\lambda_{m}}{a_{m}}E_{m}^{*},\\ I_{m}^{*} & = & \frac{\left(1-p\right)\lambda_{m}}{w_{m}}E_{m}^{*},\\ R_{m}^{*} & = & \frac{{\bar{c}}_{m}}{\mu a_{m}w_{m}}\lambda_{m}E_{m}^{*}, \end{array} \end{array}$$ (19)

and $E_{k}^{*}=A_{k}^{*}=I_{k}^{*}=R_{k}^{*}=0$ for 1 ≤ *k* ≠ *m* ≤ *n*. We can show that $S^{*},{\left\{V_{k}^{*}\right\}}_{k=1}^{n},E_{m}^{*},A_{m}^{*},I_{m}^{*},R_{m}^{*}\in T$ by using (5) to show that

$$\begin{array}{ccc} S^{*}+\sum_{k=1}^{n}V_{k}^{*}+E_{m}^{*}+A_{m}^{*}+I_{m}^{*}+R_{m}^{*} & = & \frac{(1-q)\mu }{\mu +\frac{c_{m}\lambda_{m}}{a_{m}w_{m}}E_{m}^{*}}+\sum_{k=1}^{m-1}\frac{q_{k}\mu }{\mu +\frac{c_{m}\lambda_{m}}{a_{m}w_{m}}E_{m}^{*}}+\sum_{k=m}^{n}q_{k}+E_{m}^{*}\\ & & +\frac{pw_{m}+\left(1-p\right)a_{m}}{a_{m}w_{m}}\lambda_{m}E_{m}^{*}+\frac{pw_{m}r_{m}+\left(1-p\right)a_{m}\theta_{m}}{\mu a_{m}w_{m}}\lambda_{m}E_{m}^{*}\\ & = & \left(1-q\right)+\sum_{k=1}^{n}q_{k}+E_{m}^{*}+\frac{pw_{m}+\left(1-p\right)a_{m}}{a_{m}w_{m}}\lambda_{m}E_{m}^{*}\\ & & +\frac{pw_{m}r_{m}+\left(1-p\right)a_{m}\theta_{m}}{\mu a_{m}w_{m}}\lambda_{m}E_{m}^{*}-\frac{e_{m}}{\mu }E_{m}^{*}\\ & = & 1+\frac{\lambda_{m}}{\mu }E_{m}^{*}+E_{m}^{*}-\frac{e_{m}}{\mu }E_{m}^{*}\\ & = & 1. \end{array}$$

**Remark 5.** *Theorem 2 shows that strain *m* alone persists at equilibrium if the expected number of secondary infections produced by strain *m* infected individual is greater than one. We shall later show in Theorem 8 that this equilibrium point is stable globally if* $R_{k}<1$ *for 1* ≤ k ≠ m ≤ n (*guaranteeing* $E_{k}^{*}=A_{k}^{*}=I_{k}^{*}=R_{k}^{*}=0$ *for 1* ≤ k ≠ m ≤ n) *and* $R_{m}>1$. *Theorem 2 is also valid for the case where* $\beta_{k}>{\bar{\beta }}_{k,j}$ *and* $\gamma_{k}>{\bar{\gamma }}_{k,j}$, j = *1, 2*, ⋯, k − 1. *The proof of this is shown in Theorems 17 and 18*.

Suppose $S_{2}=\left\{\tau_{1},\tau_{2}\right\}$ with strain 1 ≤ *τ*_1_ < *τ*_2_ ≤ *n*, $\tau_{j}\in Z_{+}\cup \left\{0\right\}$, *j* = 1, ⋯, 2. We denote the equilibrium point corresponding to the case where strains *τ*_1_, *τ*_2_ survive by $E_{S_{2}}$. We give theorem under which the equilibrium $E_{S_{2}}$ exists.

**Theorem 3.** *The epidemic model* (1) *has an equilibrium point $E_{S_{2}}$ in the feasible region T provided*

$$\begin{array}{c} R_{\tau_{1}}>R_{\tau_{2}}>1+\frac{R_{\tau_{1}}-1}{1+(Q_{\tau_{2}}-Q_{\tau_{1}})\frac{R_{\tau_{1}}}{1-q+Q_{\tau_{1}}}} \end{array}$$ (20)

*Proof.* Condition (20) implies that $R_{\tau_{1}}>R_{\tau_{2}}>1$. The equilibrium point $E_{S_{2}}$ is obtained as

$$\begin{array}{c} E_{S_{2}}=\left\{S^{+},V_{1}^{+},\cdots ,V_{n}^{+},E_{1}^{+},\cdots ,E_{n}^{+},A_{1}^{+},\cdots ,A_{n}^{+},I_{1}^{+},\cdots ,I_{n}^{+},R_{1}^{+},\cdots ,R_{n}^{+}\right\}, \end{array}$$ (21)

where

$$\begin{array}{c} \begin{array}{ccc} E_{\tau_{1}}^{+} & = & \frac{\mu }{e_{\tau_{1}}}\frac{R_{\tau_{1}}-R_{\tau_{2}}}{\frac{R_{\tau_{1}}}{1-q+Q_{\tau_{1}}}-\frac{R_{\tau_{2}}}{1-q+Q_{\tau_{2}}}},\\ E_{\tau_{2}}^{+} & = & \frac{\mu }{e_{\tau_{2}}}(\frac{1-q+Q_{\tau_{2}}}{R_{2}})\frac{\frac{R_{\tau_{2}}}{1-q+Q_{\tau_{2}}}+(Q_{\tau_{2}}-Q_{\tau_{1}})\frac{R_{\tau_{1}}}{1-q+Q_{\tau_{1}}}\frac{R_{\tau_{2}}}{1-q+Q_{\tau_{2}}}-\frac{R_{\tau_{1}}}{1-q+Q_{\tau_{1}}}}{\frac{R_{\tau_{1}}}{1-q+Q_{\tau_{1}}}-\frac{R_{\tau_{2}}}{1-q+Q_{\tau_{2}}}},\\ S^{+} & = & \frac{(1-q)\mu }{\mu +\sum_{j=1}^{2}(\frac{c_{\tau_{j}}\lambda_{\tau_{j}}}{a_{\tau_{j}}w_{\tau_{j}}}E_{\tau_{j}}^{+})}=\frac{1-q}{R_{\tau_{1}}},\\ V_{k}^{+} & = & \left\{ \begin{array}{c} \frac{q_{k}\mu }{\mu +\sum_{j=1}^{2}(\frac{c_{\tau_{j}}\lambda_{\tau_{j}}}{a_{\tau_{j}}w_{\tau_{j}}}E_{\tau_{j}}^{+})},\;\;\text{if}\;k\leq \tau_{1}-1,\\ \frac{q_{k}\mu }{\mu +\frac{c_{\tau_{2}}\lambda_{\tau_{2}}}{a_{\tau_{2}}w_{\tau_{2}}}E_{\tau_{2}}^{+}},\;\;\text{if}\;\;\tau_{1}\leq k<\tau_{2},\\ q_{k},\;\;\text{if}\;\;k\geq \tau_{2}, \end{array}\right.\\ A_{\tau_{k}}^{+} & = & \frac{p\lambda_{\tau_{k}}}{a_{\tau_{k}}}E_{\tau_{k}}^{+},\;\;\;k=1,2,\\ I_{\tau_{k}}^{+} & = & \frac{\left(1-p\right)\lambda_{\tau_{k}}}{w_{\tau_{k}}}E_{\tau_{k}}^{+},\;\;\;k=1,2,\\ R_{\tau_{k}}^{+} & = & \frac{{\bar{c}}_{\tau_{k}}\lambda_{\tau_{k}}}{\mu a_{\tau_{k}}w_{\tau_{k}}}E_{\tau_{k}}^{+},\;\;\;k=1,2, \end{array} \end{array}$$ (22)

and $E_{k}^{+}=A_{k}^{+}=I_{k}^{+}=R_{k}^{+}=0\;\forall \;k\neq \tau_{1},\tau_{2}$ if $R_{\tau_{1}}>R_{\tau_{2}}>1+\frac{R_{\tau_{1}}-1}{1+(Q_{\tau_{2}}-Q_{\tau_{1}})\frac{R_{\tau_{1}}}{1-q+Q_{\tau_{1}}}}$. If $R_{\tau_{1}}>R_{\tau_{2}}>1+\frac{R_{\tau_{1}}-1}{1+(Q_{\tau_{2}}-Q_{\tau_{1}})\frac{R_{\tau_{1}}}{1-q+Q_{\tau_{1}}}}$, then

$$\begin{array}{ccc} E_{\tau_{1}}^{+} & = & \frac{\mu }{e_{\tau_{1}}}\frac{R_{\tau_{1}}-R_{\tau_{2}}}{\frac{R_{\tau_{1}}}{1-q+Q_{\tau_{1}}}-\frac{R_{\tau_{2}}}{1-q+Q_{\tau_{2}}}}>0,\\ E_{\tau_{2}}^{+} & = & \frac{\mu a_{\tau_{2}}w_{\tau_{2}}}{c_{\tau_{2}}\lambda_{\tau_{2}}}\frac{\frac{R_{\tau_{2}}}{1-q+Q_{\tau_{2}}}+(Q_{\tau_{2}}-Q_{\tau_{1}})\frac{R_{\tau_{1}}}{1-q+Q_{\tau_{1}}}\frac{R_{\tau_{2}}}{1-q+Q_{\tau_{2}}}-\frac{R_{\tau_{1}}}{1-q+Q_{\tau_{1}}}}{\frac{R_{\tau_{1}}}{1-q+Q_{\tau_{1}}}-\frac{R_{\tau_{2}}}{1-q+Q_{\tau_{2}}}}\\ & = & \frac{\mu }{e_{\tau_{2}}}\frac{1}{R_{\tau_{2}}}\frac{1}{\frac{R_{\tau_{1}}}{1-q+Q_{\tau_{1}}}-\frac{R_{\tau_{2}}}{1-q+Q_{\tau_{2}}}}(\frac{1+(Q_{\tau_{2}}-Q_{\tau_{1}})\frac{R_{\tau_{1}}}{1-q+Q_{\tau_{1}}}}{1-q+Q_{\tau_{2}}})(R_{\tau_{2}}-1-\frac{R_{\tau_{1}}-1}{1+(Q_{\tau_{2}}-Q_{\tau_{1}})\frac{R_{\tau_{1}}}{1-q+Q_{\tau_{1}}}})>0. \end{array}$$

The result follows.

**Remark 6** *Since $R_{\tau_{1}}$ is non-negative, condition* (20) *implies that $R_{\tau_{1}}>R_{\tau_{2}}>1$. This seems to suggest that the system is already in endemic state with strain* τ_1_ *before the emergence of strain* τ_2_ *caused an endemic. This is also confirmed in the work of Fudolig et al* [10] *where an *SVIR* model is used to analyze the transmission of the multistrains of the COVID-19 virus. Condition* (20) *is equivalent to*

$$\begin{array}{c} R_{\tau_{1}}>R_{\tau_{2}}>\frac{bR_{\tau_{1}}}{a+\left(b-a\right)R_{\tau_{1}}}, \end{array}$$ (23)

*where* $a=1-q+Q_{\tau_{1}}$ and $b=1-q+Q_{\tau_{2}}$. *We analyze the condition geometrically in Appendix B in* S1 Appendix. *It shows that for only strains τ*_1_ *and τ*_2_ *to remain in the system on the long run, the number of infection* $R_{\tau_{2}}$ *produced by strain τ*_2_-*infected individuals must be more than* $\frac{bR_{\tau_{1}}}{a+\left(b-a\right)R_{\tau_{1}}}$ *but not up to the number* $R_{\tau_{1}}$ *produced by strain τ*_1_-*infected individuals. Once* $R_{\tau_{2}}$ *falls outside this region, then only strain τ*_1_ *or τ*_2_ *remains in the system on the long run. For instance, if* $R_{\tau_{2}}<\frac{bR_{\tau_{1}}}{a+\left(b-a\right)R_{\tau_{1}}}<R_{\tau_{1}}$, *it follows from* (22) *that the compartmental equilibrium value* $E_{\tau_{1}}^{+}>0$ *and* $E_{\tau_{2}}^{+}<0$, *so that the values* $I_{\tau_{1}}^{+}$, $A_{\tau_{1}}^{+}$, and $R_{\tau_{1}}^{+}$ *are positive but* $I_{\tau_{2}}^{+}$, $A_{\tau_{2}}^{+}$, *and* $R_{\tau_{2}}^{+}$ *are negative, showing that only strain τ*_1_ *remains on the long run. In the same sense, we can show that only one strain remains in the system on the long run if* $R_{\tau_{2}}>R_{\tau_{1}}>1$. *This endemic region is shown graphically in Appendix B in* S1 Appendix.

*Also, it can be shown that*

$$\begin{array}{c} S^{+}+\sum_{k=1}^{n}V_{k}^{+}+\sum_{j=1}^{2}(E_{\tau_{j}}^{+}+A_{\tau_{j}}^{+}+I_{\tau_{j}}^{+}+R_{\tau_{j}}^{+})=1. \end{array}$$

Suppose $S_{r}=\left\{\tau_{1},\tau_{2},\cdots ,\tau_{r}\right\}$ with strain 1 ≤ *τ*_*j* − 1_ < *τ*_*j*_ ≤ *n*, *j* = 2, ⋯, *r*, and $\tau_{1},\tau_{2},\cdots ,\tau_{r}\in Z_{+}$. We denote the equilibrium point corresponding to the case where strains *τ*_1_, *τ*_2_, ⋯, *τ*_*r*_ survive by $E_{S_{r}}$. We give theorem under which the equilibrium $E_{S_{r}}$, *r* = 2, ⋯, *n*, exists.

**Theorem 4.** *The epidemic model* (1) *has an equilibrium point $E_{S_{r}}$ in the feasible region T provided*

$$\begin{array}{c} \begin{array}{ccc} R_{\tau_{k-1}} & > & R_{\tau_{k}}>\frac{(1-q+Q_{\tau_{k}})(Q_{\tau_{k+1}}-Q_{\tau_{k-1}})\frac{R_{\tau_{k-1}}}{1-q+Q_{\tau_{k-1}}}\frac{R_{\tau_{k+1}}}{1-q+Q_{\tau_{k+1}}}}{(Q_{\tau_{k+1}}-Q_{\tau_{k}})\frac{R_{\tau_{k+1}}}{1-q+Q_{\tau_{k+1}}}+(Q_{\tau_{k}}-Q_{\tau_{k-1}})\frac{R_{\tau_{k-1}}}{1-q+Q_{\tau_{k-1}}}},\;\text{for}\;\;k=2,3,\cdots ,r-1,\\ R_{\tau_{r}} & > & 1+\frac{R_{\tau_{r-1}}-1}{1+(Q_{\tau_{r}}-Q_{\tau_{r-1}})\frac{R_{\tau_{r-1}}}{1-q+Q_{\tau_{r-1}}}}. \end{array} \end{array}$$ (24)

*Proof.* Condition (24) implies that $R_{\tau_{1}}>R_{\tau_{2}}\cdots >R_{\tau_{r}}>1$. The equilibrium point $E_{S_{r}}$ is obtained as

$$\begin{array}{c} E_{S_{r}}=\left\{S^{+},V_{1}^{+},\cdots ,V_{n}^{+},E_{1}^{+},\cdots ,E_{n}^{+},A_{1}^{+},\cdots ,A_{n}^{+},I_{1}^{+},\cdots ,I_{n}^{+},R_{1}^{+},\cdots ,R_{n}^{+}\right\}, \end{array}$$ (25)

where

$$\begin{array}{c} \begin{array}{ccc} E_{\tau_{1}}^{+} & = & \frac{\mu }{e_{\tau_{1}}}\frac{R_{\tau_{1}}-R_{\tau_{2}}}{\frac{R_{\tau_{1}}}{1-q+Q_{\tau_{1}}}-\frac{R_{\tau_{2}}}{1-q+Q_{\tau_{2}}}},\\ E_{\tau_{k}}^{+} & = & \frac{\mu }{e_{\tau_{k}}}\frac{(Q_{\tau_{k}}-Q_{\tau_{k-1}})\frac{R_{\tau_{k-1}}}{1-q+Q_{\tau_{k-1}}}\frac{R_{\tau_{k}}}{1-q+Q_{\tau_{k}}}-(Q_{\tau_{k+1}}-Q_{\tau_{k-1}})\frac{R_{\tau_{k-1}}}{1-q+Q_{\tau_{k-1}}}\frac{R_{\tau_{k+1}}}{1-q+Q_{\tau_{k+1}}}+(Q_{\tau_{k+1}}-Q_{\tau_{k}})\frac{R_{\tau_{k}}}{1-q+Q_{\tau_{k}}}\frac{R_{\tau_{k+1}}}{1-q+Q_{\tau_{k+1}}}}{\left(\frac{R_{\tau_{k-1}}}{1-q+Q_{\tau_{k-1}}}-\frac{R_{\tau_{k}}}{1-q+Q_{\tau_{k}}})(\frac{R_{\tau_{k}}}{1-q+Q_{\tau_{k}}}-\frac{R_{\tau_{k+1}}}{1-q+Q_{\tau_{k+1}}}\right)},\;\;k=2,3,\cdots ,r-1,\\ E_{\tau_{r}}^{+} & = & \frac{\mu a_{\tau_{r}}w_{\tau_{r}}}{c_{\tau_{r}}\lambda_{\tau_{r}}}\frac{\frac{R_{\tau_{r}}}{1-q+Q_{\tau_{r}}}+(Q_{\tau_{r}}-Q_{\tau_{r-1}})\frac{R_{\tau_{r}}}{1-q+Q_{\tau_{r}}}\frac{R_{\tau_{r-1}}}{1-q+Q_{\tau_{r-1}}}-\frac{R_{\tau_{r-1}}}{1-q+Q_{\tau_{r-1}}}}{\frac{R_{\tau_{r-1}}}{1-q+Q_{\tau_{r-1}}}-\frac{R_{\tau_{r}}}{1-q+Q_{\tau_{r}}}},\\ S^{+} & = & \frac{(1-q)\mu }{\mu +\sum_{j=1}^{r}(\frac{c_{\tau_{j}}\lambda_{\tau_{j}}}{a_{\tau_{j}}w_{\tau_{j}}}E_{\tau_{j}}^{+})}\\ V_{k}^{+} & = & \left\{ \begin{array}{c} =\frac{q_{k}\mu }{\mu +\sum_{j=l}^{r}(\frac{c_{\tau_{j}}\lambda_{\tau_{j}}}{a_{\tau_{j}}w_{\tau_{j}}}E_{\tau_{j}}^{+})},\;\;\text{if}\;\;\tau_{l-1}\leq k\leq \tau_{l}-1,\;\;l=1,2\cdots ,r,\;\;\tau_{0}=1,\\ q_{k},\;\;\text{if}\;\;\tau_{r}\leq k\leq n, \end{array}\right.\\ A_{\tau_{l}}^{+} & = & \frac{p\lambda_{\tau_{l}}}{a_{\tau_{l}}}E_{\tau_{l}}^{+},\;\;\;l=1,2\cdots ,r,\\ I_{\tau_{l}}^{+} & = & \frac{\left(1-p\right)\lambda_{\tau_{l}}}{w_{\tau_{l}}}E_{\tau_{l}}^{+},\;\;\;l=1,2\cdots ,r\\ R_{\tau_{l}}^{+} & = & \frac{{\bar{c}}_{\tau_{l}}}{\mu }\frac{\lambda_{\tau_{l}}}{a_{\tau_{l}}w_{\tau_{l}}}E_{\tau_{l}}^{+},\;\;\;l=1,2\cdots ,r \end{array} \end{array}$$ (26)

and $E_{k}^{+}=A_{k}^{+}=I_{k}^{+}=R_{k}^{+}=0$ for 1 ≤ *k* ≠ *τ*_1_, *τ*_2_, ⋯, *τ*_*r*_ ≤ *n* if condition (24) is satisfied. If condition (24) is satisfied, then

$$\begin{array}{ccc} E_{\tau_{1}}^{+} & > & 0,\\ E_{\tau_{r}}^{+} & = & \frac{\mu }{e_{\tau_{r}}}\frac{1}{R_{\tau_{r}}}\frac{1}{\frac{R_{\tau_{r-1}}}{1-q+Q_{\tau_{r-1}}}-\frac{R_{\tau_{r}}}{1-q+Q_{\tau_{r}}}}(\frac{1+(Q_{\tau_{r}}-Q_{\tau_{r-1}})\frac{R_{\tau_{r-1}}}{1-q+Q_{\tau_{r-1}}}}{1-q+Q_{\tau_{r}}})(R_{\tau_{r}}-1-\frac{R_{\tau_{r-1}}-1}{1+(Q_{\tau_{r}}-Q_{\tau_{r-1}})\frac{R_{\tau_{r-1}}}{1-q+Q_{\tau_{r-1}}}})>0, \end{array}$$

and for *k* = 2, 3, ⋯, *r* − 1, $\frac{R_{\tau_{k-1}}}{1-q+Q_{\tau_{k-1}}}>\frac{R_{\tau_{k}}}{1-q+Q_{\tau_{k}}}$, and $E_{\tau_{k}}^{+}>0$.

**Remark 7.** *Condition* (24) *implies*

$$\begin{array}{c} R_{\tau_{1}}>R_{\tau_{2}}>R_{\tau_{3}}>\cdots >R_{\tau_{r}}>1. \end{array}$$

*That is, the system is already in endemic state with strain τ*_*i*_ *before the emergence of strain τ*_*i* + 1_, *i* = 1, 2, ⋯, *r* − 1, *caused an endemic. Theorem 4 shows that if only strains τ*_1_, *τ*_2_, ⋯, *τ*_*r*_ *survive, then the system (without these strains) must converge to the disease-free equilibrium state*.

#### 2.4.1 Endemic equilibrium

The result for the endemic equilibrium can be calculated from Theorem 4 by extending the set $S_{r}$ to $S_{n}$. We state the result without proof in the next theorem.

**Theorem 5.** *The epidemic model* (1) *has an endemic equilibrium point $E_{S_{n}}$ given by*

$$\begin{array}{c} \begin{array}{ccc} E_{1}^{+} & = & \frac{\mu }{e_{1}}\frac{R_{1}-R_{2}}{\frac{R_{1}}{1-q}-\frac{R_{2}}{1-q+Q_{2}}},\\ E_{k}^{+} & = & \frac{\mu }{e_{k}}\frac{(Q_{k}-Q_{k-1})\frac{R_{k-1}}{1-q+Q_{k-1}}\frac{R_{k}}{1-q+Q_{k}}-(Q_{k+1}-Q_{k-1})\frac{R_{k-1}}{1-q+Q_{k-1}}\frac{R_{k+1}}{1-q+Q_{k+1}}+(Q_{k+1}-Q_{k})\frac{R_{k}}{1-q+Q_{k}}\frac{R_{k+1}}{1-q+Q_{k+1}}}{\left(\frac{R_{k-1}}{1-q+Q_{k-1}}-\frac{R_{k}}{1-q+Q_{k}})(\frac{R_{k}}{1-q+Q_{k}}-\frac{R_{k+1}}{1-q+Q_{k+1}}\right)},\;\;k=2,3,\cdots ,n-1,\\ E_{n}^{+} & = & \frac{\mu a_{n}w_{n}}{c_{n}\lambda_{n}}\frac{\frac{R_{n}}{1-q+Q_{n}}+(Q_{n}-Q_{n-1})\frac{R_{n}}{1-q+Q_{n}}\frac{R_{n-1}}{1-q+Q_{n-1}}-\frac{R_{n-1}}{1-q+Q_{n-1}}}{\frac{R_{n-1}}{1-q+Q_{n-1}}-\frac{R_{n}}{1-q+Q_{n}}},\\ S^{+} & = & \frac{(1-q)\mu }{\mu +\sum_{j=1}^{n}(\frac{c_{j}\lambda_{j}}{a_{j}w_{j}}E_{j}^{+})}\\ V_{k}^{+} & = & \left\{ \begin{array}{c} \frac{q_{k}\mu }{\mu +\sum_{j=k+1}^{n}(\frac{c_{j}\lambda_{j}}{a_{j}w_{j}}E_{j}^{+})},\;\;\text{if}\;\;j=1,2,\cdots ,n-1,\\ q_{k},\;\;\text{if}\;\;j=n, \end{array}\right.\\ A_{k}^{+} & = & \frac{p\lambda_{k}}{a_{k}}E_{k}^{+},\;\;\;k=1,2\cdots ,n,\\ I_{k}^{+} & = & \frac{\left(1-p\right)\lambda_{k}}{w_{k}}E_{k}^{+},\;\;\;k=1,2\cdots ,n\\ R_{k}^{+} & = & \frac{{\bar{c}}_{k}}{\mu }\frac{\lambda_{k}}{a_{k}w_{k}}E_{k}^{+},\;\;\;k=1,2\cdots ,n, \end{array} \end{array}$$ (27)

in the feasible region T provided

$$\begin{array}{c} \begin{array}{ccc} R_{\tau_{k-1}} & > & R_{\tau_{k}}>\frac{(1-q+Q_{\tau_{k}})(Q_{\tau_{k+1}}-Q_{\tau_{k-1}})\frac{R_{\tau_{k-1}}}{1-q+Q_{\tau_{k-1}}}\frac{R_{\tau_{k+1}}}{1-q+Q_{\tau_{k+1}}}}{(Q_{\tau_{k+1}}-Q_{\tau_{k}})\frac{R_{\tau_{k+1}}}{1-q+Q_{\tau_{k+1}}}+(Q_{\tau_{k}}-Q_{\tau_{k-1}})\frac{R_{\tau_{k-1}}}{1-q+Q_{\tau_{k-1}}}},\;for\;\;k=2,3,\cdots ,n-1,\\ R_{\tau_{n}} & > & 1+\frac{R_{\tau_{n-1}}-1}{1+(Q_{\tau_{n}}-Q_{\tau_{n-1}})\frac{R_{\tau_{n-1}}}{1-q+Q_{\tau_{n-1}}}}. \end{array} \end{array}$$ (28)

In the next section, we discuss the convergence of the system $\tilde{y}$ in (4). We study conditions under which all strain infections are eradicated in the population on the long run. We also discuss the condition under which certain strain of the disease persists.

## 3 Stability analysis

In this section, we discuss the convergence of system (1) under certain conditions. That is, we study condition(s) under which the system converges to disease-free $E_{0}$ or equilibriums $E_{S_{r}}$, *r* = 1, 2, ⋯, *n*.

### 3.1 Stability analysis of the disease-free equilibrium point

**Theorem 6.** *The disease-free equilibrium $E_{0}$ is locally asymptotically stable in the feasible region* T *if* $R_{0}<1$.

Given the initial condition ${\tilde{y}}_{0}\in T$, Theorem 6 shows that if the system $\tilde{y}$ satisfying (1) starts near the initial point ${\tilde{y}}_{0}$, then the system converges to the equilibrium point $E_{0}$ (that is, all the strain of infections are eradicated) provided the threshold $R_{0}<1$. We use the idea presented in [27] to prove Theorem 6.

*Proof.* Let $I_{n\times n}$ be the n × n identity matrix, $y=\tilde{y}-E_{0}$, ***β*** = {β_*1*_, ⋯, β_n_}, **γ** = {γ_*1*_, ⋯, γ_n_}, the matrices $U_{q}\left(\beta \right)$ and $U_{q}\left(\gamma \right)$ defined by

$$\begin{array}{ccc} U_{q}\left(\beta \right) & = & \left(\begin{array}{cccccc} 0 & q_{1}{\bar{\beta }}_{2,1} & q_{1}{\bar{\beta }}_{3,1} & q_{1}{\bar{\beta }}_{4,1} & \cdots & q_{1}{\bar{\beta }}_{n,1}\\ 0 & 0 & q_{2}{\bar{\beta }}_{3,2} & q_{2}{\bar{\beta }}_{4,2} & \cdots & q_{2}{\bar{\beta }}_{n,2}\\ 0 & 0 & 0 & q_{3}{\bar{\beta }}_{4,3} & \cdots & q_{3}{\bar{\beta }}_{n,3}\\ \vdots & \vdots & \vdots & \ddots & \ddots & \vdots \\ \vdots & \vdots & \vdots & \vdots & \ddots & q_{n-1}{\bar{\beta }}_{n,n-1}\\ 0 & 0 & \cdots & 0 & 0 & 0 \end{array}),\;\;U_{q}\left(\gamma \right)=(\begin{array}{cccccc} 0 & q_{1}{\bar{\gamma }}_{2,1} & q_{1}{\bar{\gamma }}_{3,1} & q_{1}{\bar{\gamma }}_{4,1} & \cdots & q_{1}{\bar{\gamma }}_{n,1}\\ 0 & 0 & q_{2}{\bar{\gamma }}_{3,2} & q_{2}{\bar{\gamma }}_{4,2} & \cdots & q_{2}{\bar{\gamma }}_{n,2}\\ 0 & 0 & 0 & q_{3}{\bar{\gamma }}_{4,3} & \cdots & q_{3}{\bar{\gamma }}_{n,3}\\ \vdots & \vdots & \vdots & \ddots & \ddots & \vdots \\ \vdots & \vdots & \vdots & \vdots & \ddots & q_{n-1}{\bar{\gamma }}_{n,n-1}\\ 0 & 0 & \cdots & 0 & 0 & 0 \end{array}\right) \end{array}$$

so that

$$\begin{array}{ccc} G & = & (\begin{array}{ccc} 0_{1\times n} & -\left(1-q\right)U_{q}\left(\gamma \right) & -\left(1-q\right)U_{q}\left(\beta \right) \end{array}),\\ H & = & (\begin{array}{ccc} 0_{n\times n} & rI_{n\times n} & \theta I_{n\times n} \end{array}),\\ A & = & (\begin{array}{ccc} M(e) & -M(\tilde{\gamma }) & -M(\tilde{\beta })\\ -pM(\lambda ) & M(a) & 0_{n\times n}\\ -\left(1-p\right)M(\lambda ) & 0_{n\times n} & M(w) \end{array}), \end{array}$$

where ***M*** is defined in (9). The linearization of the model (1) at the equilibrium point $E_{0}$ is derived as

$$\begin{array}{c} y^{{}^{\prime }}=Ay, \end{array}$$ (29)

where

$$\begin{array}{c} A=(\begin{array}{cccc} -\mu & 0_{1\times n} & H & 0_{1\times n}\\ 0_{n\times 1} & -\mu I_{n\times n} & G & 0_{n\times n}\\ 0_{3n\times 1} & 0_{3n\times n} & -A & 0_{3n\times n}\\ 0_{n\times 1} & 0_{n\times n} & H & -\mu I_{n\times n} \end{array}). \end{array}$$

In order to show that the equilibrium point $E_{0}$ is locally stable, we need to show that the maximum real part, *s*(**A**), of **A** is negative. From the structure of the matrix **A**, this reduces to showing that the maximum real part of the eigenvalues of matrix -A is negative (or equivalently, s(A)>0). The determinant det(A) of the matrix A is

$$\begin{array}{c} \text{det}\left(A\right)=\prod_{k=1}^{n}(1-R_{k}). \end{array}$$

If $R_{0}<1$, then det(A)>0. Matrix A is a non-singular *Z* matrix that can be written in the form

$$\begin{array}{c} A=LU, \end{array}$$ (30)

where L and U are lower and upper diagonal matrices, respectively, with positive diagonals obtained as

$$\begin{array}{ccc} L_{i,j} & = & \frac{1}{D_{j}}|\begin{array}{cccc} A_{1,1} & A_{1,2} & \cdots & A_{1,j}\\ A_{2,1} & A_{2,2} & \cdots & A_{2,j}\\ \vdots & \vdots & \cdots & \vdots \\ A_{j-1,1} & A_{j-1,2} & \cdots & A_{j-1,j}\\ A_{i,1} & A_{i,2} & \cdots & A_{i,j} \end{array}|,\;\;\text{for}\;\;i\geq j\neq 1,\;\;L_{i,1}=\frac{|A_{i,1}|}{D_{1}}\;\;\text{for}\;i=1,2,...,3n,\;\;\text{and}\;0\;\;\text{elsewhere},\\ U_{i,j} & = & \frac{1}{D_{i-1}}|\begin{array}{cccc} A_{1,1} & \cdots & A_{1,i-1} & A_{1,j}\\ A_{2,1} & \cdots & A_{2,i-1} & A_{2,j}\\ \vdots & \vdots & \vdots & \vdots \\ A_{i,1} & \cdots & A_{i,i-1} & A_{i,j} \end{array}|,\;\;\text{for}\;\;1\neq i\leq j,\;\;U_{1,j}=A_{1,j},\;\text{for}\;\;j=1,2,...,3n,\;\text{and}\;\;0\;\;\text{elsewhere}, \end{array}$$

where $D_{0}:=1$, and $D_{j}=|\begin{array}{cccc} A_{1,1} & A_{1,2} & \ldots & A_{1,j}\\ A_{2,1} & A_{2,2} & \ldots & A_{2,j}\\ \vdots & \vdots & \ldots & \vdots \\ A_{j,1} & A_{j,2} & \ldots & A_{j,j} \end{array}|$ for *j* = 1, 2, …, 3*n*, with

$$\begin{array}{ccc} D_{j} & = & \prod_{k=1}^{j}e_{k},\;\;\text{if}\;\;1\leq j\leq n,\\ D_{n+j} & = & (\prod_{l=1}^{n}e_{l})\prod_{k=1}^{j}[a_{k}(1-R_{k}+\left(1-p\right)(\left(1-q\right)\beta_{k}+\sum_{l=1}^{k-1}{\bar{\beta }}_{k,l}q_{l})a_{k}\lambda_{k})],\;\;\text{if}\;\;1\leq j\leq n,\\ D_{2n+j} & = & \prod_{l=1}^{j}(a_{l}e_{l}w_{l}(1-R_{l}))\prod_{k=j+1}^{n}[a_{k}e_{k}(1-R_{k}+\left(1-p\right)(\left(1-q\right)\beta_{k}+\sum_{l=1}^{k-1}{\bar{\beta }}_{k,l}q_{l})a_{k}\lambda_{k})],\;\;\text{if}\;\;1\leq j\leq n-1,\\ \;D_{3n} & = & \prod_{l=1}^{n}a_{l}e_{l}w_{l}(1-R_{l}). \end{array}$$

The diagonals $L_{j,j}=1$ and $U_{i,j}=D_{j}/D_{j-1}>0$ if $R_{0}<1$. Therefore, the matrix A is a non-singular *M* matrix, and hence, a *P*-matrix. It follows from Berman [54] and Plemmons [53] that the maximum real part of the eigenvalues of -A is negative.

**Remark 8.** *Theorem 6 shows that if the population starts sufficiently close to ${\bar{y}}_{0}$, then the system $\bar{y}$ converges to $E_{0}$ if $R_{0}<1$. That is, the proportion of the susceptible size converges to 1* − q, *the proportion of the size of the vaccinated class immune to strain *k* converges to the vaccination rate *q*_*k*_ of individuals with strain *k* of infection, and no infected class, hence no need of recovery on the long run if $R_{0}<1$.*

We prove in the next theorem that the population, irrespective of where it starts from, converges to the point $E_{0}$ if $R_{0}\leq 1$.

**Theorem 7.** *The disease-free equilibrium* $E_{0}$ *is globally stable in the feasible region* T *if* $R_{0}\leq 1$.

*Proof.* Define the Lyapunov function V by

$$\begin{array}{ccc} \bar{V} & = & \left(S-S^{0}-S^{0}\;\text{ln}\frac{S}{S^{0}})+\sum_{k=1}^{n}{\bar{\varphi }}_{k}(V_{k}-V_{k}^{0}-V_{k}^{0}\;\text{ln}\frac{V_{k}}{V_{k}^{0}})+\sum_{k=1}^{n}({\bar{z}}_{k}E_{k}+{\bar{f}}_{k}I_{k}+{\bar{g}}_{k}A_{k}\right) \end{array}$$

where

$$\begin{array}{ccc} {\bar{z}}_{k} & = & R_{k},\\ {\bar{\varphi }}_{k} & = & 1,\\ {\bar{f}}_{k} & = & \frac{1}{w_{k}}(S^{0}\beta_{k}+\sum_{j=1}^{k}{\bar{\phi }}_{j-1}V_{j-1}^{0}\beta_{k})\\ {\bar{g}}_{k} & = & \frac{1}{a_{k}}(S^{0}\gamma_{k}+\sum_{j=1}^{k}{\bar{\phi }}_{j-1}V_{j-1}^{0}\gamma_{k}),\;\;\text{for}\;\text{all}\;k=1,2,\cdots ,n, \end{array}$$

where ${\bar{\phi }}_{0}=0$. Let *s* = *S*/*S*^0^, $v_{k}=V_{k}/V_{k}^{0}$. If $R_{k}\leq 1$, it follows that

$$\begin{array}{ccc} d\bar{V}/dt & = & \left(1-q\right)\mu -\mu \left(S-S^{0}\right)-\mu \left(1-q\right)S^{0}/S+\sum_{k=1}^{n}{\bar{\phi }}_{k}\mu (q_{k}+V_{k}^{0})\\ & & +\sum_{j=1}^{n}\left({\bar{z}}_{j}-1\right)(\beta_{j}I_{j}+\gamma_{j}A_{j})S-\sum_{k=1}^{n-1}\sum_{j=k+1}^{n}\left({\bar{\phi }}_{k}-{\bar{z}}_{k}\right)(\beta_{j}I_{j}+\gamma_{j}A_{j})V_{k}-\mu \sum_{k=1}^{n}{\bar{\phi }}_{k}(V_{k}+q_{k}V_{k}^{0}/V_{k})\\ & & +\sum_{k=1}^{n}(S^{0}\beta_{k}+\sum_{j=1}^{k}{\bar{\phi }}_{j-1}V_{j-1}^{0}\beta_{k}-{\bar{f}}_{k}w_{k})I_{k}+\sum_{k=1}^{n}(S^{0}\gamma_{k}+\sum_{j=1}^{k}{\bar{\phi }}_{j-1}V_{j-1}^{0}\gamma_{k}-{\bar{g}}_{k}a_{k})A_{k}\\ & & +\sum_{k=1}^{n}(\left(1-p\right){\bar{f}}_{k}\lambda_{k}+p{\bar{g}}_{k}\lambda_{k}-{\bar{z}}_{k}e_{k})E_{k}\\ & \leq & -\mu S^{0}(s+\frac{1}{s}-2)-\sum_{k=1}^{n}\mu {\bar{\phi }}_{k}q_{k}(v_{k}+\frac{1}{v_{k}}-2) \end{array}$$

where

$$\begin{array}{ccc} S^{0}\beta_{k}+\sum_{j=1}^{k-1}{\bar{\phi }}_{j}V_{j}^{0}{\bar{\beta }}_{k,j}-{\bar{f}}_{k}w_{k} & = & 0,\\ S^{0}\gamma_{k}+\sum_{j=1}^{k}{\bar{\phi }}_{j-1}V_{j-1}^{0}\gamma_{k}-{\bar{g}}_{k}a_{k} & = & 0,\\ \left(1-p\right){\bar{f}}_{k}\lambda_{k}+p{\bar{g}}_{k}\lambda_{k}-z_{k}e_{k} & = & \frac{1}{a_{k}w_{k}}(\left(1-q\right)c_{k}\lambda_{k}+\sum_{j=1}^{k}\phi_{j-1}(q_{j-1}c_{k}\lambda_{k})-z_{k}a_{k}e_{k}w_{k})\\ & = & e_{k}(R_{k}-z_{k})=0, \end{array}$$

and

$$\begin{array}{c} 2(\mu S^{0}+\sum_{k=1}^{n}\mu {\bar{\phi }}_{k}q_{k})=\left(1-q\right)\mu +\mu S^{0}+\sum_{k=1}^{n}{\bar{\phi }}_{k}\mu (q_{k}+V_{k}^{0})=2\mu \end{array}$$

using (6). Using the fact that the arithmetic mean of a list of non-negative real numbers is greater than or equal to the geometric mean of the same list [55], it follows that if $R_{0}\leq 1$, then $d\bar{V}/dt\leq 0$. Also, $d\bar{V}/dt=0$ if *S* = *S*^0^ = 1 − *q*, *I*_*k*_ = *E*_*k*_ = *R*_*k*_ = 0, $V_{k}=V_{k}^{0}=q_{k}$ (or equivalently, if *s* = 1, *I*_*k*_ = *E*_*k*_ = *R*_*k*_ = 0, *v*_*k*_ = 1) for all *k* = 1, 2, ⋯, *n*. Since $E_{0}$ is the largest invariant set in the subset of T where $d\bar{V}/dt=0$, its global stability follows by the LaSalle’s Invariance Principle [56]

**Remark 9.** *Theorem 7 shows that the susceptible class converges to a fraction 1* − q *of the entire population size (this is simply the population size without the vaccinated group), the vaccinated class immune to strain k converges to a fraction* q_k_ *of population size, and no infected class, hence no need of recovery on the long run if* $R_{0}\leq 1$. *This suggests the threshold for disease eradication is the number* $R_{0}$.

### 3.2 Bound for the critical vaccination threshold

Herd immunity is a state where significant proportion of the population is immune to an infection so that only few susceptible individuals can be infected and transmit the infection [57]. Classical vaccine-induced herd-immunity threshold suggests that the spread of a disease can be stopped by vaccinating certain fraction of the population. This might be invalid and biased due to many factors such as emergence of multi variant strains of the virus/disease, the dynamic nature of virus transmission, presence of immunity due to infection, changes in implementation and adherence to public health measures, and uncertainties in vaccine effectiveness and duration of immunity [57, 41]. These can cause the estimation of the Herd-immunity threshold to be imprecise. In this section, we aim to estimate a bound for the minimum proportion of the population that must be vaccinated in order for certain infection to die out in the population.

Let E_*j*_ ∈ (0, 1] denotes the proportion of vaccinated individuals against strain *j* who are protected by vaccines, for *j* = 1, 2, ⋯, *n*. It follows from Remark 4 that we can write $R_{k}$ in (14) in terms of $R_{c,k}$ in (17) as follows:

$$\begin{array}{c} R_{j}=\left(1-c_{j}E_{j}\right)R_{c,j}, \end{array}$$ (31)

for some constant *c*_*j*_ ∈ (0, 1). If the vaccine is perfect and provides 100% immunity, then the vaccine effectiveness E_*j*_ = 1, otherwise, E_*j*_ < 1 if it provides only partial immunity. According to Theorem 7, the population reaches herd immunity with respect to strain *j*, with incidence of the infection declining, if $R_{j}\leq 1$. This yield

$$\begin{array}{c} c_{j}\geq (1-\frac{1}{R_{c,j}})\frac{1}{E_{j}}. \end{array}$$

The number

$$\begin{array}{c} H_{j}=(1-\frac{1}{R_{c,j}})\frac{1}{E_{j}} \end{array}$$ (32)

is the minimum proportion of a population infected that must be vaccinated to stop strain *j* from spreading. This number is referred to as the herd immunity threshold [41]. In the presence of multiple vaccines for strains *j* = 1, 2, ⋯, *n* with effectiveness E_1_, E_2_, ⋯, E_*n*_, respectively, let *H*_*m*_ and *H*_*M*_ denote the minimum $\min_{1\leq j\leq n}H_{j}$ and maximum $\max_{1\leq j\leq n}H_{j}$ herd immunity thresholds for the strains *j* = 1, 2, ⋯, *n*, respectively. The interval

$$\begin{array}{c} \left[H_{m},H_{M}\right] \end{array}$$ (33)

contains the maximum proportion of the population expected to be vaccinated to stop the spread of the disease.

**Remark 10.** *Caveats to the estimate*

We note here that the bound in (33) is calculated for a population satisfying the dynamics given in (1). The bound is expected to change in the emergence of a new strain of the virus. Also, the bound is calculated without taking into consideration the proportion of those who have immunity from the virus. These and more are some of the caveats to this estimates.

Sometimes new variants emerge and disappear. Other times, new variants persist. We study conditions under which new variant, say strain *m*, persists in the population. In the next section, we study the behavior of the system in the case where disease is not completely eradicated in the system.

### 3.3 Stability analysis of strain *m* equilibrium

In the next theorem, we study how the population behaves on the long run if the number, $R_{m}$, of new infections produced by infected individual with strain *m* is greater than one, while $R_{k}\leq 1$ for 1 ≤ *k* ≠ *m* ≤ *n*.

**Theorem 8.** *The strain* m *equilibrium* $E_{m}$ *for the epidemic model* (1) *is globally stable in the feasible region* T *if* $R_{k}\leq 1$ *for all 1* ≤ k ≠ m ≤ n *and* $R_{m}>1$.

*Proof.* According to Theorem 2, the strain *m* equilibrium $E_{m}$ exists and non-negative if $R_{m}>1$. Define the Lyapunov function V by

$$\begin{array}{ccc} V & = & \left(S-S^{*}-S^{*}\;\text{ln}\;\frac{S}{S^{*}})+z_{m}(E_{m}-E_{m}^{*}-E_{m}^{*}\;\text{ln}\;\frac{E_{m}}{E_{m}^{*}})+f_{m}(I_{m}-I_{m}^{*}-I_{m}^{*}\;\text{ln}\;\frac{I_{m}}{I_{m}^{*}})+g_{m}(A_{m}-A_{m}^{*}-A_{m}^{*}\;\text{ln}\;\frac{A_{m}}{A_{m}^{*}}\right)\\ & & +d_{m}(R_{m}-R_{m}^{*}-R_{m}^{*}\;\text{ln}\;\frac{R_{m}}{R_{m}^{*}})+\sum_{k=1}^{n}\varphi_{k}(V_{k}-V_{k}^{*}-V_{k}^{*}\;\text{ln}\;\frac{V_{k}}{V_{k}^{*}})+\sum_{k=1\;k\neq m}^{n}(z_{k}E_{k}+f_{k}I_{k}+g_{k}A_{k}+d_{k}R_{k}) \end{array}$$

where

$$\begin{array}{ccc} z_{k} & = & \varphi_{k}=d_{k}=1,\\ f_{k} & = & \frac{1}{w_{k}}(S^{*}\beta_{k}+\sum_{j=1}^{k}\varphi_{j-1}V_{j-1}^{*}\beta_{k}),\\ g_{k} & = & \frac{1}{a_{k}}(S^{*}\gamma_{k}+\sum_{j=1}^{k}\varphi_{j-1}V_{j-1}^{*}\gamma_{k}),\;\;\text{for}\;\text{all}\;k=1,2,\cdots ,n, \end{array}$$

with *φ*_0_ = 0. Let *s* = *S*/S^*^, $v_{k}=V_{k}/V_{k}^{*}$, $\sum_{m}=E_{m}/E_{m}^{*}$, $\tau_{m}=I_{m}/I_{m}^{*}$, $\Delta_{m}=A_{m}/A_{m}^{*}$. It follows that

$$\begin{array}{ccc} dV/dt & = & \left(1-q\right)\mu -\mu \left(S-S^{*}\right)-\mu \left(1-q\right)S^{*}/S+z_{m}e_{m}E_{m}^{*}+\sum_{k=1}^{n}\varphi_{k}\mu (q_{k}+V_{k}^{*})+f_{m}w_{m}I_{m}^{*}+g_{m}a_{m}A_{m}^{*}\\ & & +\sum_{j=1}^{n}\left(z_{j}-1\right)(\beta_{j}I_{j}+\gamma_{j}A_{j})S-\sum_{k=1}^{n-1}\sum_{j=k+1}^{n}\left(\varphi_{k}-z_{k}\right)(\beta_{j}I_{j}+\gamma_{j}A_{j})V_{k}-\left(1-p\right)f_{m}\lambda_{m}I_{m}^{*}E_{m}/I_{m}\\ & & -pg_{m}\lambda_{m}A_{m}^{*}E_{m}/A_{m}-z_{m}E_{m}^{*}((\beta_{m}I_{m}/E_{m}+\gamma_{m}A_{m}/E_{m})S+\sum_{k=1}^{m-1}V_{k}(\beta_{m}I_{m}/E_{m}+\gamma_{m}A_{m}/E_{m}))\\ & & -\mu \sum_{k=1}^{n}\varphi_{k}(V_{k}+q_{k}V_{k}^{*}/V_{k})+\sum_{k=1}^{n}(S^{*}\beta_{k}+\sum_{j=1}^{k}\varphi_{j-1}V_{j-1}^{*}\beta_{k}-f_{k}w_{k})I_{k}+\sum_{k=1}^{n}(S^{*}\gamma_{k}+\sum_{j=1}^{k}\varphi_{j-1}V_{j-1}^{*}\gamma_{k}-g_{k}a_{k})A_{k}\\ & & +\sum_{k=1}^{n}(\left(1-p\right)f_{k}\lambda_{k}+pg_{k}\lambda_{k}-z_{k}e_{k})E_{k}\\ & \leq & -\eta (s+\frac{1}{s}-2)-u(\frac{s\tau_{m}}{\Sigma_{m}}+\frac{\Sigma_{m}}{\tau_{m}}+\frac{1}{s}-3)-b(\frac{s\Delta_{m}}{\Sigma_{m}}+\frac{\Sigma_{m}}{\Delta_{m}}+\frac{1}{s}-3)-\sum_{k=1}^{m-1}\sigma_{k}(\frac{v_{k}\tau_{m}}{\Sigma_{m}}+\frac{\Sigma_{m}}{\tau_{m}}+\frac{1}{v_{k}}-3)\\ & & -\sum_{k=1}^{m-1}\varphi_{k}(\frac{v_{k}\Delta_{m}}{\Sigma_{m}}+\frac{\Sigma_{m}}{\Delta_{m}}+\frac{1}{v_{k}}-3)-\sum_{k=1}^{n}{ϒ}_{k}(v_{k}+\frac{1}{v_{k}}-2) \end{array}$$

if $R_{m}>1$ and $R_{k}\leq 1$ for 1 ≤ *k* ≠ *m* ≤ *n*, where

$$\begin{array}{ccc} \left(1-p\right)f_{k}\lambda_{k}+pg_{k}\lambda_{k}-z_{k}e_{k} & = & \frac{1}{a_{k}w_{k}}(S^{*}c_{k}\lambda_{k}+\sum_{j=1}^{k-1}(\varphi_{j}V_{j}^{*}{\bar{c}}_{k,j}\lambda_{k})-a_{k}e_{k}w_{k}z_{k})\\ & & \left\{ \begin{array}{c} =0,\;\;if\;\;k=m,\;\;using\;(19),\\ \leq e_{k}(R_{k}-1)\leq 0,\;\;if\;\;R_{k}\leq 1,\;\;k\neq m, \end{array}\right. \end{array}$$

$$\begin{array}{ccc} \eta & = & \mu S^{*},\\ u & = & z_{m}\beta_{m}I_{m}^{*}S^{*},\\ b & = & z_{m}\gamma_{m}A_{m}^{*}S^{*},\\ \sigma_{k} & = & z_{m}\beta_{m}I_{m}^{*}V_{k}^{*},\;\;for\;\;1\leq k\leq m-1\\ \varphi_{k} & = & z_{m}\gamma_{m}A_{m}^{*}V_{k}^{*},\;\;for\;\;1\leq k\leq m-1\\ {ϒ}_{k} & = & \mu \varphi_{k}V_{k}^{*},\;\;for\;\;1\leq k\leq n \end{array}$$

and

$$\begin{array}{ccc} 2(\eta +\sum_{k=1}^{n}{ϒ}_{k})+3(u+b+\sum_{k=1}^{m-1}(\sigma_{k}+\varphi_{k})) & = & \left(1-q\right)\mu +\mu S^{*}+f_{m}w_{m}I_{m}^{*}+g_{m}a_{m}A_{m}^{*}\\ & & +z_{m}e_{m}E_{m}^{*}+\mu \sum_{j=1}^{n}\varphi_{k}(q_{k}+V_{k}^{*}) \end{array}$$

using (19). Using the fact that the arithmetic mean of a list of non-negative real numbers is greater than or equal to the geometric mean of the same list [56], it follows that dV/dt≤0. Equality holds if *S* = S^*^, *I*_*k*_ = *E*_*k*_ = *R*_*k*_ = 0 for all *k* ≠ *m*, $V_{k}=V_{k}^{*}$ for all *k* = 1, 2, ⋯, *n*, $I_{m}/I_{m}^{*}=E_{m}/E_{m}^{*}=R_{m}/R_{m}^{*}=1$. Since $E_{m}$ is the largest invariant set in the subset of T where dV/dt=0, its global stability follows by the LaSalle’s Invariance Principle [56].

**Remark 11**. *Theorem 8 shows that the system* $\bar{y}$ *converges to* $E_{m}$ *on the long run if* $R_{k}\leq 1$ *for 1* ≤ k ≠ m ≤ n *and* $R_{m}>1$ *irrespective of the starting point. That is, if the number*, $R_{m}$, *of new infections is greater than one while* $R_{k}\leq 1$ *for 1* ≤ k ≠ m ≤ n, *then on the long run, the susceptible class converges to a fraction* $S^{*}=\frac{\left(1-q\right)}{R_{m}}<1-q$ *of the entire population size, no exposure to any strain other than strain* m *in the population (hence no infection other than those caused by strain* m, *and no need for recovery), and before the existence of strain* m (*that is*, k < m), *the vaccinated class immune to strain* k < m *converges to a fraction* $V_{k}^{*}=\frac{q_{k}}{R_{m}}<q_{k}$ *of the population size. We see from Theorem 7 that if there is no endemic in the population, the susceptible population S converges on the long run to* (1 − q) × *100% of the population. This number reduces by* $(1-\frac{1}{R_{m}})\times 100\%$ *in the presence of an emerging strain* m. *Also, from Theorem 7, the vaccinated class V*_k_ *converges to q*_*k*_ × *100% of the population size if there is no endemic in the population. This number also reduces by* $(1-\frac{1}{R_{m}})\times 100\%$ *in the presence of an emerging strain* m. *If k* > m *on the other hand, since* $R_{k}\leq 1$ *for* *1* ≤ *k* ≠ m ≤ n, *we have the vaccinated class immune to strain* k (*k* > m) *converging to a fraction* $V_{k}^{*}=q_{k}$ *of the population, no exposure to any strain other than strain m in the population (hence no infection and no need for recovery)*.

### 3.4 Stability analysis of the equilibrium point $E_{S_{2}}$

We give the proof of the stability of the equilibrium point $E_{S_{2}}$ in the next theorem.

**Theorem 9.** *The equilibrium point* $E_{S_{2}}$ *is globally stable in the feasible region* T *if* $R_{k}<1$ *for 1* ≤ k ≠ τ_*1*_, τ_*2*_ ≤ n *and condition* (20) *is satisfied*.

*Proof.* *Assume* $R_{k}\leq 1$ *for all* k ∉ τ_*1*_, τ_*2*_ *and condition* (20) *is satisfied. The existence of the equilibrium point* $E_{S_{2}}$ *follows from Theorem 3. Define the Lyapunov function* $V_{2}$ *by*

$$\begin{array}{ccc} V_{2} & = & \left(S-S^{+}-S^{+}\;\;\text{ln}\;\frac{S}{S^{+}})+\sum_{l=1}^{2}z_{\tau_{l}}(E_{\tau_{l}}-E_{\tau_{l}}^{+}-E_{\tau_{l}}^{+}\;\;\text{ln}\;\frac{E_{\tau_{l}}}{E_{\tau_{l}}^{+}})+\sum_{l=1}^{2}f_{\tau_{l}}(I_{\tau_{l}}-I_{\tau_{l}}^{+}-I_{\tau_{l}}^{+}\;\;\text{ln}\;\frac{I_{\tau_{l}}}{I_{\tau_{l}}^{+}}\right)\\ & & +\sum_{l=1}^{2}g_{\tau_{l}}(A_{\tau_{l}}-A_{\tau_{l}}^{+}-A_{\tau_{l}}^{+}\;\;\text{ln}\;\frac{A_{\tau_{l}}}{A_{\tau_{l}}^{+}})+\sum_{k=1}^{n}\varphi_{k}(V_{k}-V_{k}^{+}-V_{k}^{+}\;\;\text{ln}\;\frac{V_{k}}{V_{k}^{+}})+\sum_{\begin{array}{c} k=1\\ k\notin \tau_{1},\tau_{2} \end{array}}^{n}(z_{k}E_{k}+f_{k}I_{k}+g_{k}A_{k}+d_{k}R_{k}) \end{array}$$

where

$$\begin{array}{c} \begin{array}{ccc} z_{k} & = & \varphi_{k}=d_{k}=1,\\ f_{k} & = & \frac{1}{w_{k}}(S^{+}\beta_{k}+\sum_{j=1}^{k}\varphi_{j-1}V_{j-1}^{+}\beta_{k}),\\ g_{k} & = & \frac{1}{a_{k}}(S^{+}\gamma_{k}+\sum_{j=1}^{k}\varphi_{j-1}V_{j-1}^{+}\gamma_{k}),\;\;\text{for}\;\text{all}\;k=1,2,\cdots ,n, \end{array} \end{array}$$ (34)

with *φ*_0_ = 0. The derivative of $V_{2}$ computed along the solution of (1) is

$$\begin{array}{ccc} dV_{2}/dt & = & \left(1-q\right)\mu -\mu \left(S-S^{+}\right)-\mu \left(1-q\right)S^{+}/S+\sum_{j=1}^{2}(z_{\tau_{j}}e_{\tau_{j}}E_{\tau_{j}}^{+}+f_{\tau_{j}}w_{\tau_{j}}I_{\tau_{j}}^{+}+g_{\tau_{j}}a_{\tau_{j}}A_{\tau_{j}}^{+})+\sum_{k=1}^{n}\varphi_{k}\mu (q_{k}+V_{k}^{+})\\ & & +\sum_{j=1}^{n}\left(z_{j}-1\right)(\beta_{j}I_{j}+\gamma_{j}A_{j})S-\sum_{k=1}^{n-1}\sum_{j=k+1}^{n}\left(\varphi_{k}-z_{k}\right)(\beta_{j}I_{j}+\gamma_{j}A_{j})V_{k}-\left(1-p\right)\sum_{j=1}^{2}f_{\tau_{j}}\lambda_{\tau_{j}}I_{\tau_{j}}^{+}E_{\tau_{j}}/I_{\tau_{j}}\\ & & -p\sum_{j=1}^{2}g_{\tau_{j}}\lambda_{\tau_{j}}A_{\tau_{j}}^{+}E_{\tau_{j}}/A_{\tau_{j}}-\sum_{j=1}^{2}z_{\tau_{j}}E_{\tau_{j}}^{+}((\beta_{\tau_{j}}I_{\tau_{j}}/E_{\tau_{j}}+\gamma_{\tau_{j}}A_{\tau_{j}}/E_{\tau_{j}})S+\sum_{k=1}^{\tau_{j}-1}V_{k}(\beta_{\tau_{j}}I_{\tau_{j}}/E_{\tau_{j}}+\gamma_{\tau_{j}}A_{\tau_{j}}/E_{\tau_{j}}))\\ & & -\mu \sum_{k=1}^{n}\varphi_{k}(V_{k}+q_{k}V_{k}^{+}/V_{k})+\sum_{k=1}^{n}(S^{+}\beta_{k}+\sum_{j=1}^{k-1}\varphi_{j}V_{j}^{+}\beta_{k}-f_{k}w_{k})I_{k}+\sum_{k=1}^{n}(S^{+}\gamma_{k}+\sum_{j=1}^{k-1}\varphi_{j}V_{j}^{+}\gamma_{k}-g_{k}a_{k})A_{k}\\ & & +\sum_{k=1}^{n}(\left(1-p\right)f_{k}\lambda_{k}+pg_{k}\lambda_{k}-z_{k}e_{k})E_{k} \end{array}$$

It follows from (34) and Remark 6 that $R_{\tau_{1}}>R_{\tau_{2}}>1$ and

$$\begin{array}{ccc} S^{+}\beta_{k}+\sum_{j=1}^{k-1}\varphi_{j}V_{j}^{+}\beta_{k}-f_{k}w_{k} & = & 0,\\ S^{+}\gamma_{k}+\sum_{j=1}^{k-1}\varphi_{j}V_{j}^{+}\gamma_{k}-g_{k}a_{k} & = & 0,\\ \left(1-p\right)f_{k}\lambda_{k}+pg_{k}\lambda_{k}-z_{k}e_{k} & = & \frac{1}{a_{k}w_{k}}(S^{+}c_{k}\lambda_{k}+\sum_{j=1}^{k-1}(\varphi_{j}V_{j}^{+}c_{k}\lambda_{k})-a_{k}e_{k}w_{k}z_{k})\\ & & \left\{ \begin{array}{c} =0,\;\;if\;\;k\in S_{2},\;\;using\;\left(22\right),\\ \leq e_{k}(R_{k}-1)\leq 0,\;\;if\;\;R_{k}\leq 1,\;\;k\notin S_{2}, \end{array}\right. \end{array}$$

Let *s* = *S*/*S*^+^, $v_{k}=V_{k}/V_{k}^{+}$, $\sum_{\tau_{l}}=E_{\tau_{l}}/E_{\tau_{l}}^{+}$, $\tau_{\tau_{l}}=I_{\tau_{l}}/I_{\tau_{l}}^{+}$, $\Delta_{\tau_{l}}=A_{\tau_{l}}/A_{\tau_{l}}^{+}$. Define

$$\begin{array}{ccc} \eta & = & \mu S^{+},\\ u_{\tau_{j}} & = & z_{\tau_{j}}\beta_{\tau_{j}}I_{\tau_{j}}^{+}S^{+},\\ b_{\tau_{j}} & = & z_{\tau_{j}}\gamma_{\tau_{j}}A_{\tau_{j}}^{+}S^{+},\\ \sigma_{k,\tau_{j}} & = & z_{\tau_{j}}\beta_{\tau_{j}}I_{\tau_{j}}^{+}V_{k}^{+},\;\;for\;\;1\leq k\leq \tau_{j}-1,\\ \varphi_{k,\tau_{j}} & = & z_{\tau_{j}}\gamma_{\tau_{j}}A_{\tau_{j}}^{+}V_{k}^{+},\;\;for\;\;1\leq k\leq \tau_{j}-1,\\ {ϒ}_{k} & = & \mu \varphi_{k}V_{k}^{+},\;\;for\;\;1\leq k\leq n. \end{array}$$

We have

$$\begin{array}{ccc} dV_{2}/dt & \leq & -\eta (s+\frac{1}{s}-2)-\sum_{j=1}^{2}u_{\tau_{j}}(\frac{s\tau_{\tau_{j}}}{\Sigma_{\tau_{j}}}+\frac{\Sigma_{\tau_{j}}}{\tau_{\tau_{j}}}+\frac{1}{s}-3)-\sum_{j=1}^{2}b_{\tau_{j}}(\frac{s\Delta_{\tau_{j}}}{\Sigma_{\tau_{j}}}+\frac{\Sigma_{\tau_{j}}}{\Delta_{\tau_{j}}}+\frac{1}{s}-3)\\ & & -\sum_{j=1}^{2}\sum_{k=1}^{\tau_{j}-1}\sigma_{k,\tau_{j}}(\frac{v_{k}\tau_{\tau_{j}}}{\Sigma_{\tau_{j}}}+\frac{\Sigma_{\tau_{j}}}{\tau_{\tau_{j}}}+\frac{1}{v_{k}}-3)-\sum_{j=1}^{2}\sum_{k=1}^{\tau_{j}-1}\varphi_{k,\tau_{j}}(\frac{v_{k}\Delta_{\tau_{j}}}{\Sigma_{\tau_{j}}}+\frac{\Sigma_{\tau_{j}}}{\Delta_{\tau_{j}}}+\frac{1}{v_{k}}-3)-\sum_{k=1}^{n}{ϒ}_{k}(v_{k}+\frac{1}{v_{k}}-2), \end{array}$$

where

$$\begin{array}{ccc} 2(\eta +\sum_{k=1}^{n}{ϒ}_{k})+3\sum_{j=1}^{2}(u_{\tau_{j}}+b_{\tau_{j}}+\sum_{k=1}^{\tau_{j}-1}(\sigma_{k,\tau_{j}}+\varphi_{k,\tau_{j}})) & = & \left(1-q\right)\mu +\mu S^{+}+\sum_{j=1}^{2}z_{\tau_{j}}e_{\tau_{j}}E_{\tau_{j}}^{+}+\mu \sum_{j=1}^{n}\varphi_{k}(q_{k}+V_{k}^{+})\\ & & +\sum_{j=1}^{2}(f_{\tau_{j}}w_{\tau_{j}}I_{\tau_{j}}^{+}+g_{\tau_{j}}a_{\tau_{j}}A_{\tau_{j}}^{+}) \end{array}$$

using (22). It follows that $dV_{2}/dt\leq 0$. Equality holds if *S* = *S*^+^, *I*_*k*_ = *E*_*k*_ = *R*_*k*_ = 0 for all $k\notin S_{2}$, $V_{k}=V_{k}^{+}$ for all *k* = 1, 2, ⋯, *n*, $I_{\tau_{j}}/I_{\tau_{j}}^{+}=E_{\tau_{j}}/E_{\tau_{j}}^{+}=R_{\tau_{j}}/R_{\tau_{j}}^{+}=1$. Since $E_{S_{2}}$ is the largest invariant set in the subset of T where $dV_{2}/dt=0$, its global stability follows by the LaSalle’s Invariance Principle [56].

### 3.5 Stability analysis of the equilibrium point $E_{S_{r}}$

**Theorem 10.** *For r* = 3, 4, ⋯, *n*, *the equilibrium point* $E_{S_{r}}$ *is globally stable in the feasible region* T *if* $R_{k}\leq 1$ *for all* $k\notin S_{r}$ *and condition* (24) *is satisfied*.

*Proof.* The proof of Theorem 10 is similar to that of Theorem 9 by extending $S_{2}$ to $S_{r}$, *r* = 3, 4, ⋯, *n*.

**Remark 12.** *Theorem 10 can be extended to a case where* $S_{r}=S_{n}$.

## 4 Model for re-infected recovered and vaccinated population

There have been confirmed cases of the COVID-19 reinfections around the world [38, 40, 42]. In this section, in addition to assuming that individuals vaccinated against strain *k* can gets infected with emerging strains *j* > *k*, we also discuss the case where individuals who recovered from strain *k* can be infected with emerging strains *j* > *k*. For this additional assumption, we extend (1) to the form

$$\begin{array}{c} \begin{array}{ccc} dS & = & (\left(1-q\right)\mu -S\sum_{j=1}^{n}(\beta_{j}I_{j}+\gamma_{j}A_{j})-\mu S)dt,\;\;S\left(t_{0}\right)=S_{0},\\ dV_{k} & = & (q_{k}\mu -V_{k}\sum_{j=k+1}^{n}(\beta_{j}I_{j}+\gamma_{j}A_{j})-\mu V_{k})dt,\;\;V_{k}\left(t_{0}\right)=V_{k0},\;\;k=1,2,\cdots ,n-1\\ dV_{n} & = & (q_{n}\mu -\mu V_{n})dt,\;\;V_{n}\left(t_{0}\right)=V_{n0},\\ dE_{1} & = & (S(\beta_{1}I_{1}+\gamma_{1}A_{1})-\left(\mu +\lambda_{1}\right)E_{1})\;dt,\;\;E_{1}\left(t_{0}\right)=E_{10},\\ dE_{k} & = & (S(\beta_{k}I_{k}+\gamma_{k}A_{k})+\sum_{j=1}^{k-1}V_{j}(\beta_{k}I_{k}+\gamma_{k}A_{k})+\sum_{j=1}^{k-1}R_{j}(\beta_{k}I_{k}+\gamma_{k}A_{k})-\left(\mu +\lambda_{k}\right)E_{k})\;dt,\;\;E_{k}\left(t_{0}\right)=E_{k0},\;\;k=2,3,\cdots ,n\\ dA_{k} & = & (p\lambda_{k}E_{k}-\left(\mu +r_{k}\right)A_{k})\;dt,\;\;k=1,2,\cdots ,n\;\;\;A_{k}\left(t_{0}\right)=A_{k0},\;\;k=1,2,\cdots ,n\\ dI_{k} & = & (\left(1-p\right)\lambda_{k}E_{k}-\left(\mu +\theta_{k}\right)I_{k})\;dt,\;\;I_{k}\left(t_{0}\right)=I_{k0},\;\;k=1,2,\cdots ,n,\\ dR_{k} & = & (\theta_{k}I_{k}+r_{k}A_{k}-R_{k}\sum_{j=k+1}^{n}(\beta_{j}I_{j}+\gamma jA_{j})-\mu R_{k})dt,\;\;R_{k}\left(t_{0}\right)=R_{k0},\;\;k=1,2,\cdots ,n-1\\ dR_{n} & = & (\theta_{n}I_{n}+r_{n}A_{n}-\mu R_{n})\;dt,R_{n}\left(t_{0}\right)=R_{n0}, \end{array} \end{array}$$ (35)

**Fig 2:**
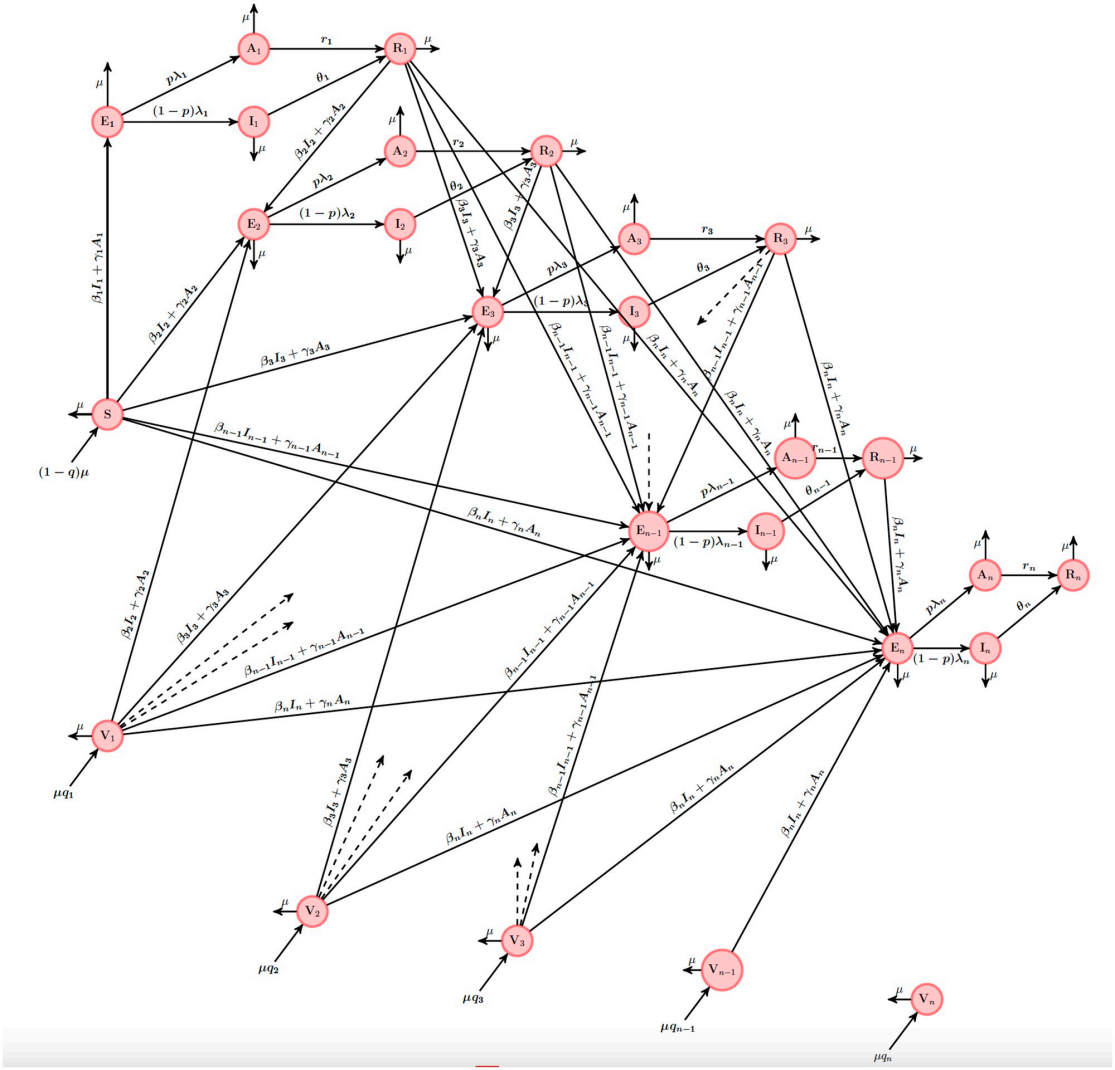
Schematic diagram for the epidemic model (35). The circle compartments represent group of individuals.

The schematic diagram of model (35) is given in Fig 2.

### 4.1 Validity of the epidemic model (35)

In this section, we discuss the validity of the proposed epidemic model (35).

**Theorem 11.** *If S*_0_ > 0, *V*_*k*0_ > 0, *E*_*k*0_ > 0, *A*_*k*0_ > 0, *I*_*k*0_ > 0, *R*_*k*0_ > 0, *then there exist a positive unique solution of* (35) *in the feasible region* T *for all t* ≥ 0.

*Proof.* The proof is similar to Theorem 1.

### 4.2 Existence of equilibrium points for model (35)

The disease-free equilibrium of the system (35) is the same as that of the system (1), and given by

$$\begin{array}{c} E_{0}=\left\{S^{0}=1-q,V_{1}^{0}=q_{1},\cdots ,V_{n}^{0}=q_{n},E_{1}^{0}=0,\cdots ,E_{n}^{0}=0,A_{1}^{0}=0,\cdots ,A_{n}^{0}=0,I_{1}^{0}=0,\cdots ,I_{n}^{0}=0,R_{1}^{0}=0,\cdots ,R_{n}^{0}=0\right\}. \end{array}$$ (36)

### 4.3 Reproduction number for model (35)

It can also be shown in a similar manner that model (35) has the same reproduction numbers $R_{k}$ and $R_{0}$ as that of model (1). This shows that the number of infection in a population where every individual who recovered from strain *k* is immune to all possible strains is the same for the population where individuals who recovered from strain *k* can still be infected with emerging strains *j* > *k*. Hence, for strain *k* infected individual, the expected number, $R_{k}$ of new infections produced by individual with strain *k* in a susceptible population satisfying (35) is the same as (11). Likewise, the reproduction number $R_{0}$ for the system (35) is obtained in a similar manner to (14).

### 4.4 Existence of endemic equilibrium points for model (35)

**Theorem 12**. *The strain *m* unique equilibrium point $E_{m}$ for the epidemic model* (35) *exists in the feasible region T provided $R_{m}>1$. The value of $E_{m}$ is the same for models* (1) *and* (35) *and obtained in Theorem (2)*.

**Remark 13**. *Theorem 12 shows that the existence of a single strain endemic (strain m in this case) does not depend on whether the recovered class is immune to the strain or not. Regardless of the immunity status of the recovered strain k-class to the strain, the equilibrium point of the system will be* $E_{m}$ *if* $R_{m}>1$.

Define

$$\begin{array}{c} {\bar{R}}_{\tau_{k}}=(1-q+Q_{k})\frac{{\bar{c}}_{\tau_{k}}\lambda_{\tau_{k}}}{a_{\tau_{k}}e_{\tau_{k}}w_{\tau_{k}}},\;\;\;k=1,2,\cdots ,n. \end{array}$$ (37)

It can be shown that ${\bar{R}}_{\tau_{k}}<1-q+Q_{k}$. Conditions for existence of other equilibrium points are given in Theorem 13.

**Theorem 13**. *The epidemic model* (35) *has an equilibrium point* $E_{S_{2}}$ *satisfying*

$$\begin{array}{c} \begin{array}{ccc} E_{\tau_{1}}^{+} & = & \frac{\mu }{e_{\tau_{1}}}\frac{R_{\tau_{1}}-R_{\tau_{2}}}{\frac{R_{\tau_{1}}}{1-q+Q_{\tau_{1}}}-\frac{R_{\tau_{2}}}{1-q+Q_{\tau_{2}}}(1-\frac{{\bar{R}}_{\tau_{1}}}{1-q+Q_{\tau_{1}}})},\\ E_{\tau_{2}}^{+} & = & \frac{\mu }{e_{\tau_{2}}}(\frac{1-q+Q_{\tau_{2}}}{R_{\tau_{2}}})\frac{\frac{R_{\tau_{2}}}{1-q+Q_{\tau_{2}}}+(Q_{\tau_{2}}-Q_{\tau_{1}})\frac{R_{\tau_{1}}}{1-q+Q_{\tau_{1}}}\frac{R_{\tau_{2}}}{1-q+Q_{\tau_{2}}}-\frac{R_{\tau_{1}}}{1-q+Q_{\tau_{1}}}+\frac{R_{\tau_{2}}}{1-q+Q_{\tau_{2}}}\frac{{\bar{R}}_{\tau_{1}}}{1-q+Q_{\tau_{1}}}(R_{\tau_{1}}-1)}{\frac{R_{\tau_{1}}}{1-q+Q_{\tau_{1}}}-\frac{R_{\tau_{2}}}{1-q+Q_{\tau_{2}}}(1-\frac{{\bar{R}}_{\tau_{1}}}{1-q+Q_{\tau_{1}}})},\\ S^{+} & = & \frac{1-q}{R_{\tau_{1}}},\\ V_{k}^{+} & = & \left\{ \begin{array}{c} \frac{q_{k}}{R_{\tau_{1}}},\;\;\text{if}\;k\leq \tau_{1}-1\\ \frac{q_{k}\mu }{\mu +\frac{c_{\tau_{2}}\lambda_{\tau_{2}}}{a_{\tau_{2}}w_{\tau_{2}}}E_{\tau_{2}}^{+}},\;\;\text{if}\;\;\tau_{1}\leq k<\tau_{2},\\ q_{k},\;\;\text{if}\;\;k\geq \tau_{2}, \end{array}\right.\\ A_{\tau_{k}}^{+} & = & \frac{p\lambda_{\tau_{k}}}{a_{\tau_{k}}}E_{\tau_{k}}^{+},\;\;\;k=1,2,\\ I_{\tau_{k}}^{+} & = & \frac{\left(1-p\right)\lambda_{\tau_{k}}}{w_{\tau_{k}}}E_{\tau_{k}}^{+},\;\;\;k=1,2\\ R_{\tau_{1}}^{+} & = & \frac{{\bar{c}}_{\tau_{1}}\lambda_{\tau_{1}}}{a_{\tau_{1}}w_{\tau_{1}}}\frac{E_{\tau_{1}}^{+}}{\mu +\frac{c_{\tau_{2}}\lambda_{\tau_{2}}}{a_{\tau_{2}}w_{\tau_{2}}}E_{\tau_{2}}^{+}},\\ R_{\tau_{2}}^{+} & = & \frac{{\bar{c}}_{\tau_{2}}\lambda_{\tau_{2}}}{\mu a_{\tau_{2}}w_{\tau_{2}}}E_{\tau_{2}}^{+}, \end{array} \end{array}$$ (38)

$E_{k}^{+}=A_{k}^{+}=I_{k}^{+}=R_{k}^{+}=0$ *for k* ≠ *τ*_1_, *τ*_2_, *in the feasible region* T *provided*

$$\begin{array}{c} R_{\tau_{1}}>R_{\tau_{2}}>1+\frac{R_{\tau_{1}}-1}{1+\frac{Q_{\tau_{2}}-Q_{\tau_{1}}+{\bar{R}}_{\tau_{1}}}{1-q+Q_{\tau_{1}}-{\bar{R}}_{\tau_{1}}}R_{\tau_{1}}} \end{array}$$ (39)

*is satisfied*.

*Proof*. The proof is similar to that of Theorem 4.

**Remark 14** *Unlike Remark 13, we see here that the existence of two endemic equilibrium points depends on the immunity status of the recovered class. The exposed and infectious equilibrium points* $E_{\tau_{k}}^{+}$, $A_{\tau_{k}}^{+}$, *and* $I_{\tau_{k}}^{+}$ *for model* (35) *are greater compared to that of model* (1). *This is because unlike model* (1), *model* (35) *suggests that the recovered population R*_*k*_ *is not fully immune to emerging strains* *j* > *k*, *increasing the possibility of populating the exposed and infected classes. In addition, condition* (39) *implies that* $R_{\tau_{1}}>R_{\tau_{2}}>1$. *The condition is equivalent to* $R_{\tau_{1}}>R_{\tau_{2}}>\frac{bR_{\tau_{1}}}{a+\left(b-a\right)R_{\tau_{1}}}$, *where* $a=1-q+Q_{\tau_{1}}-{\bar{R}}_{\tau_{1}}$ *and* $b=1-q+Q_{\tau_{2}}$.

### 4.5 Global analysis of equilibrium points for model (35)

**Theorem 14** *The disease-free equilibrium $E_{0}$ for model* (35) *is locally asymptotically stable in the feasible region T if $R_{0}<1$ and globally stable in the feasible region if $R_{0}\leq 1$.*

*Proof*. The proof is similar to that given in Theorems 6 and 7.

**Theorem 15**. *The strain m equilibrium* $E_{m}$ *for the epidemic model* (35) *is globally stable in the feasible region* T if $R_{k}\leq 1$ *for all* 1 ≤ *k* ≠ *m* ≤ *n* *and* $R_{m}>1$.

*Proof*. The proof is similar to the proof of Theorem 8.

**Theorem 16**. *The equilibrium point* $E_{S_{2}}$ *for model* (35) *is globally stable in the feasible region* T if $R_{k}<1$ *for 1* ≤ *k* ≠ *τ*_1_, *τ*_2_ ≤ *n and condition* (39) *is satisfied.*

*Proof*. The proof is similar to the proof of Theorem 9.

## 5 Results: Covid-19 data analysis

We apply models (1) and (35) to analyze the United States daily COVID-19 cases (number of infection cases, recovery cases, and vaccination). The daily COVID-19 cases data are available on the CDC website [58] for the COVID-19 periods 04/01/2020 till present. We analyze the confirmed COVID-19 infection cases, and vaccination cases as reported by U.S. states, U.S. territories, New York City, and the District of Columbia from the previous day. Since the two recent variant of concerns (VOC) in the United States are the Delta and Omicron variants, we consider the case where *n* = 2 using model (35), with the Delta variant as Strain *τ*_1_ = 1 and the Omicron variant as strain *τ*_2_ = 2. Two analyses are performed in this section. The first analysis is shown in Section 5.1 to confirm the validity of the results derived in this work. Using published and estimated COVID-19 parameters, we confirm the stability results for the disease-free equilibrium, strain 1 equilibrium, strain 2 equilibrium, and the endemic equilibrium $E_{S_{2}}$. In section 5.2, the real COVID-19 cases for the United States is analyzed using model (35).

### 5.1 Simulation results using published and estimated parameters

Using model (35), we confirm the existence, and stability of the disease-free equilibrium, strain 1 equilibrium, strain 2 equilibrium, and the endemic equilibrium $E_{S_{2}}$ for the case where strains 1 and 2 represent the Delta and Omicron variants, respectively. The CDC data for vaccination shows that about 63% of the population of the United States are fully vaccinated (either taken the two dozes of Pfizer or Moderna, or the single dose of Jannsen) as at January 25, 2022. For this reason, we chose *q*_1_ and *q*_2_ to be in the interval [0, 0.63]. In their paper, Saldana [59] shows that about 80% of infection was asymptomatic with average incubation and recovery time of 5 and 10 days, respectively. Also, Hay et al. [37] shows that the mean duration of Delta and Omicron’s infections is 10.9 days and 9.87 days, with 95% confidence intervals (8.83, 10.9) and (9.41, 12.4), respectively. For this reason, we set *r*_1_, *θ*_1_ ∈ [1/10.9, 1/8.83] and *r*_2_, *θ*_2_ ∈ [1/12.4, 1/9.41]. Based on the five COVID-19 Pandemic Planning Scenarios estimated by CDC, the number of infections that are asymptomatic is uncertain and in the interval [0.15, 0.7], with the best estimate of 30%. We set *μ* = 0.0124. Bernal et al. [60] shows in their studies that the effectiveness of two doses of BNT162b2 (Pfizer) vaccines was 88.0% (95% CI, 85.3 to 90.1) among those with the delta variant. On November 16, 2020, the company Moderna announced that their vaccine is more than 94% effective at preventing COVID-19, based on an analysis of 95 cases [61]. We use these estimates for the Herd immunity plot. Following results from Jing et al, we select values for the incubation period to be between 2 days and 7 days, so that λ_1_, λ_2_ ∈ [1/7, 1/2]. The range of parameters used in the simulation is shown in Table 3.

**Table 3:** Model parameter values.

Parameter	Units	Range	References
*β*_1_	day^−1^	[0.1, 1.4007]	[37, 62–66]
*β*_2_	day^−1^	[0.1, 1.6761]	Assumed
*γ*_1_	day^−1^	[0.07, 0.9567]	[62, 63, 65, 66]
*γ*_2_	day^−1^	[0.07, 0.9567]	Assumed
*q*_1_	day^−1^	[0, 0.63]	CDC
*q*_2_	day^−1^	[0, 0.63]	CDC
*p*	day^−1^	[0.4, 0.6]	CDC
λ_1_	day^−1^	[1/7, 1/2]	[67]
λ_2_	day^−1^	[1/7, 1/2]	[67]
*r*_1_	day^−1^	[1/10.9, 1/8.83]	[37]
*r*_2_	day^−1^	[1/12.4, 1/9.41]	[37]
*θ*_1_	day^−1^	[1/10.9, 1/8.83]	[37]
*θ*_2_	day^−1^	[1/12.4, 1/9.41]	[37]

**Fig 3:**
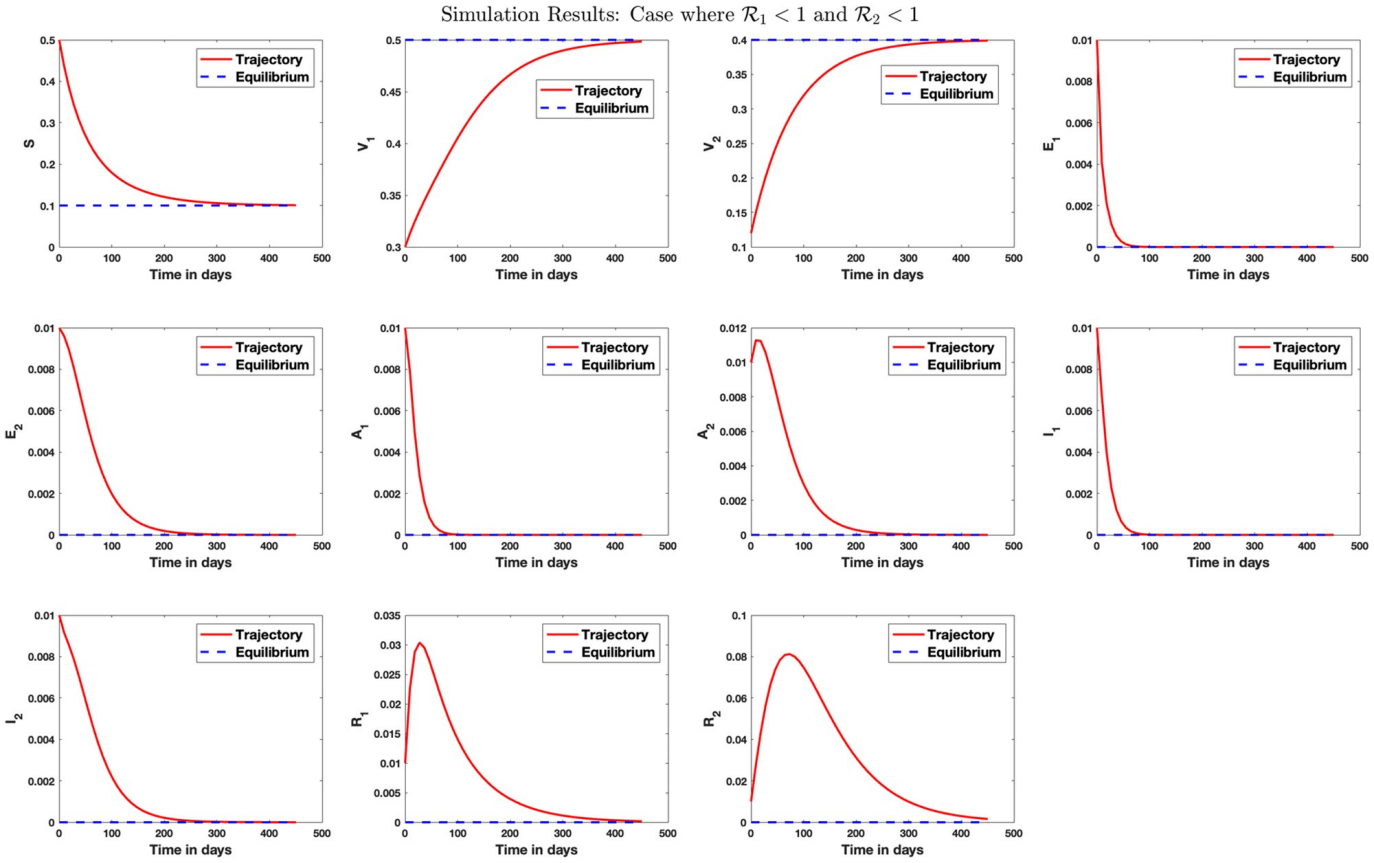
Stability analysis for the disease-free equilibrium. Case where $R_{1}<1$ and $R_{2}<1$. Here, we set *β*_1_ = 0.07, *β*_2_ = 0.2, *γ*_1_ = 0.1, *γ*_2_ = 0.12, *μ* = 1/80.3, *q*_1_ = 0.5, *q*_2_ = 0.4, λ_1_ = 1/5, λ_2_ = 1/4, *r*_1_ = 1/10.5, *r*_2_ = 1/9, *θ*_1_ = 0.09, *θ*_2_ = 0.1. The reproduction numbers $R_{1}=0.08$ and $R_{2}=0.74$ so that $R_{0}=0.74$. On the long run, the susceptible population reduces to 10% of the population size while the population receiving vaccination against strains 1 and 2 increases to 50% and 40% of the population size, respectively, in this scenario. The exposed and infected population size converges to zero on the long run. The disease-free equilibrium is calculated as $E_{0}=\left\{0.1,0.5,0.4,0,0,0,0,0,0,0,0\right\}$.

Simulation result for the case where the population is free of disease on the long run is shown in Fig 3.

**Fig 4:**
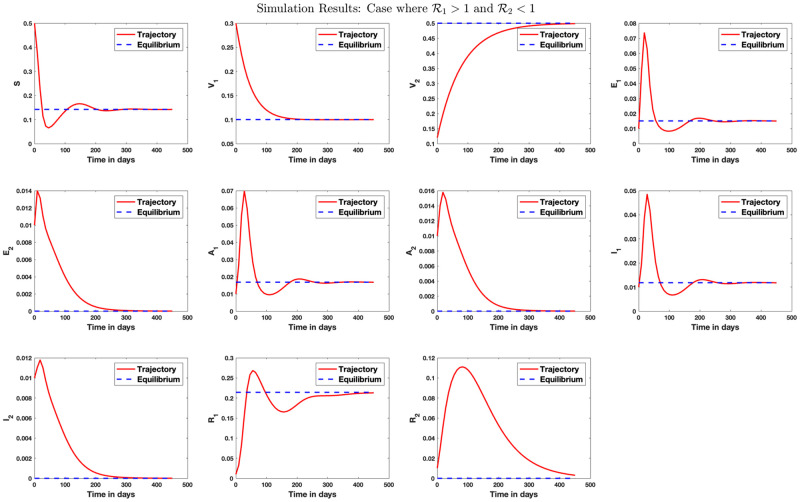
Stability analysis for the strain 1 equilibrium. Case where $R_{1}>1$ and $R_{2}<1$. This is a case where *β*_1_ = 1.2, *β*_2_ = 0.4, *γ*_1_ = 0.5, *γ*_2_ = 0.08, *μ* = 1/80.3, *q*_1_ = 0.1, *q*_2_ = 0.5, λ_1_ = 1/5, λ_2_ = 1/4, *r*_1_ = 1/10.5, *r*_2_ = 1/9, *θ*_1_ = 0.09, *θ*_2_ = 0.1. A scenario where the population of those receiving vaccination against strain 1 (with high transmission rate) dropped, leading to strain 1 endemic. In this case, $R_{1}=2.81$ and $R_{2}=0.86$ so that $R_{0}=2.81$. The strain 1 equilibrium is obtained as $E_{1}=\left\{0.1422,0.1000,0.5000,0.0151,0,0.0168,0,0.0118,0,0.2141,0\right\}.$

**Fig 5:**
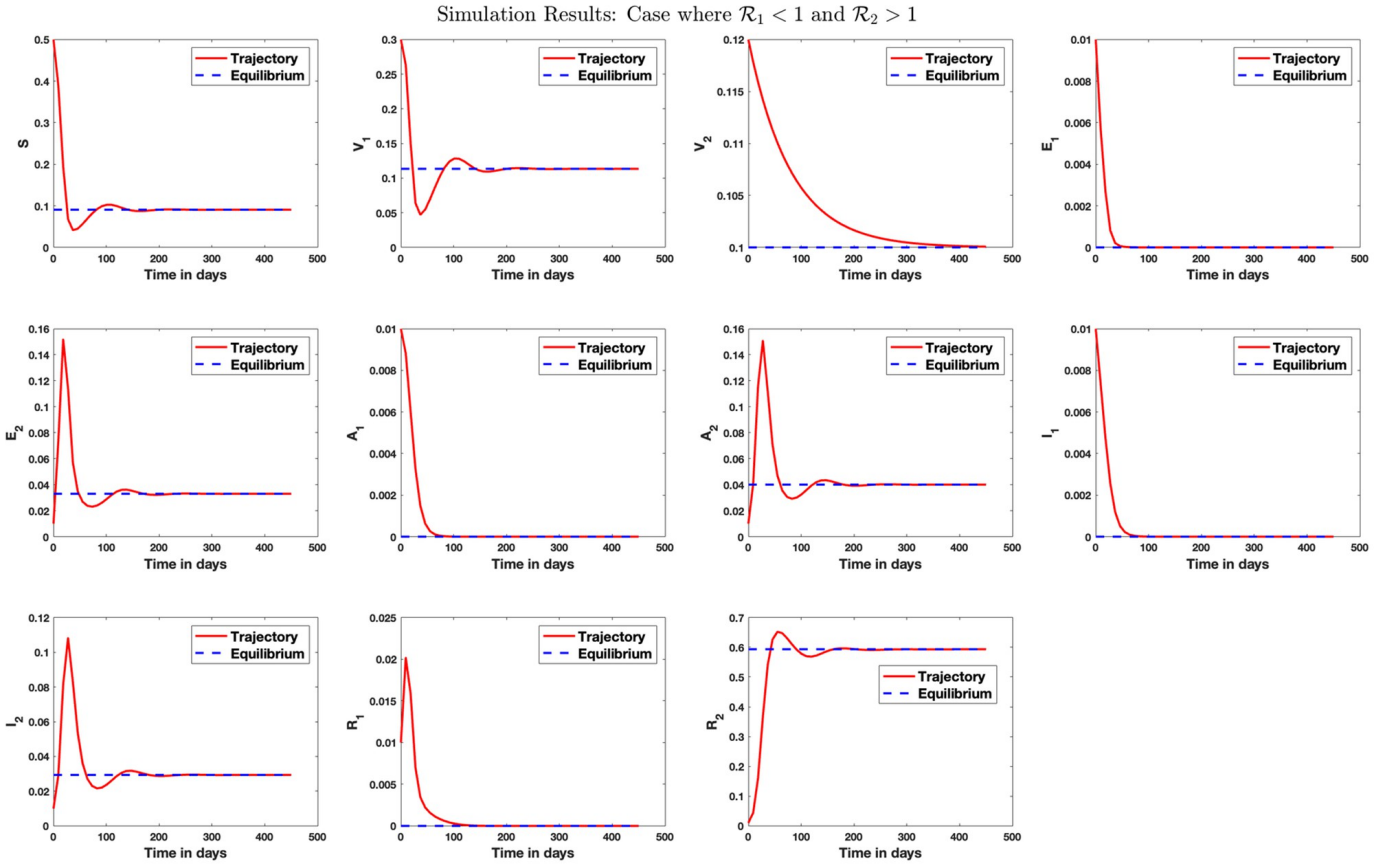
Stability analysis for strain 2 equilibrium. Case where $R_{1}<1$ and $R_{2}>1$. In this case, we set *β*_1_ = 0.18, *β*_2_ = 0.9, *γ*_1_ = 0.1, *γ*_2_ = 0.4, *μ* = 1/80.3, *q*_1_ = 0.5, *q*_2_ = 0.1, λ_1_ = 1/5, λ_2_ = 1/4, *r*_1_ = 1/10.5, *r*_2_ = 1/9, *θ*_1_ = 0.09, *θ*_2_ = 0.1. In this case, $R_{1}=0.47$ and $R_{2}=4.41$ so that $R_{0}=4.41$. The strain 2 equilibrium is obtained as $E_{2}=\left\{0.0907,0.1134,0.1000,0,0.0330,,0,0.0401,0,0.0294,0,0.5934\right\}$. This vector shows proportions that each of the population sizes *S*, *V*_1_, *V*_2_, *E*_1_, *E*_2_, *A*_1_, *A*_2_, *I*_1_, *I*_2_, *R*_1_, *R*_2_ converge to on the long run.

**Fig 6:**
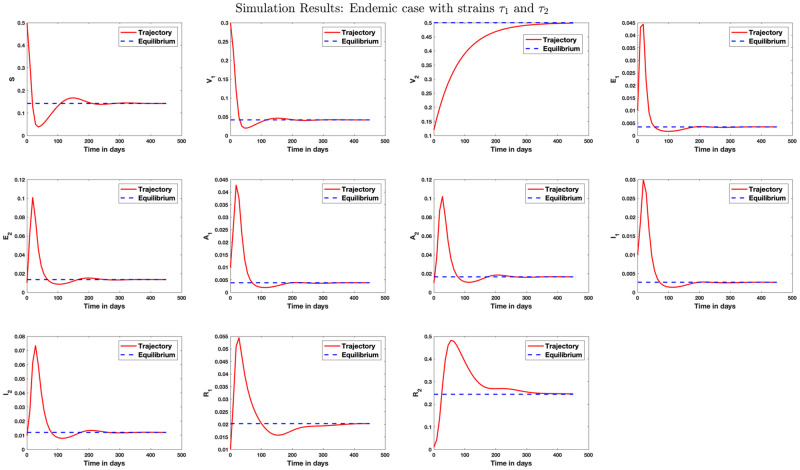
Stability analysis for the equilibrium $E_{S_{2}}$. Case where $R_{1}>R_{2}>1+\frac{R_{1}-1}{1+\frac{Q_{2}-Q_{1}+{\bar{R}}_{1}}{1-q+Q_{1}-{\bar{R}}_{1}}R_{1}}$. In this case, we set *β*_1_ = 1.2, *β*_2_ = 0.9, *γ*_1_ = 0.5, *γ*_2_ = 0.4, *μ* = 1/80.3, *q*_1_ = 0.1, *q*_2_ = 0.5, λ_1_ = 1/5, λ_2_ = 1/4, *r*_1_ = 1/10.5, *r*_2_ = 1/9, *θ*_1_ = 0.09, *θ*_2_ = 0.1. In this case, $R_{1}=2.81$ and $R_{2}=2.45$ so that $R_{0}=2.81$. We see in this case that $1+\frac{R_{1}-1}{1+\frac{Q_{2}-Q_{1}+{\bar{R}}_{1}}{1-q+Q_{1}-{\bar{R}}_{1}}R_{1}}=1.10$ and $R_{1}>R_{2}>1+\frac{R_{1}-1}{1+\frac{Q_{2}-Q_{1}+{\bar{R}}_{1}}{1-q+Q_{1}-{\bar{R}}_{1}}R_{1}}$, implying the existence of endemic with more than one strain. We see an endemic with both strains 1 and 2 because the population is already in an endemic state with strain 1 before strain 2 caused an endemic, and the number of secondary infection caused by strain 2 is more than $1+\frac{R_{1}-1}{1+\frac{Q_{2}-Q_{1}+{\bar{R}}_{1}}{1-q+Q_{1}-{\bar{R}}_{1}}R_{1}}$ but not up to that caused by strain 1. The endemic equilibrium in this case is $E_{S_{2}}=\left\{0.1422,0.0416,0.5000,0.0034,0.0136,0.0038,0.0165,0.0027,0.0121,0.0203,0.2439\right\}.$

**Fig 7:**
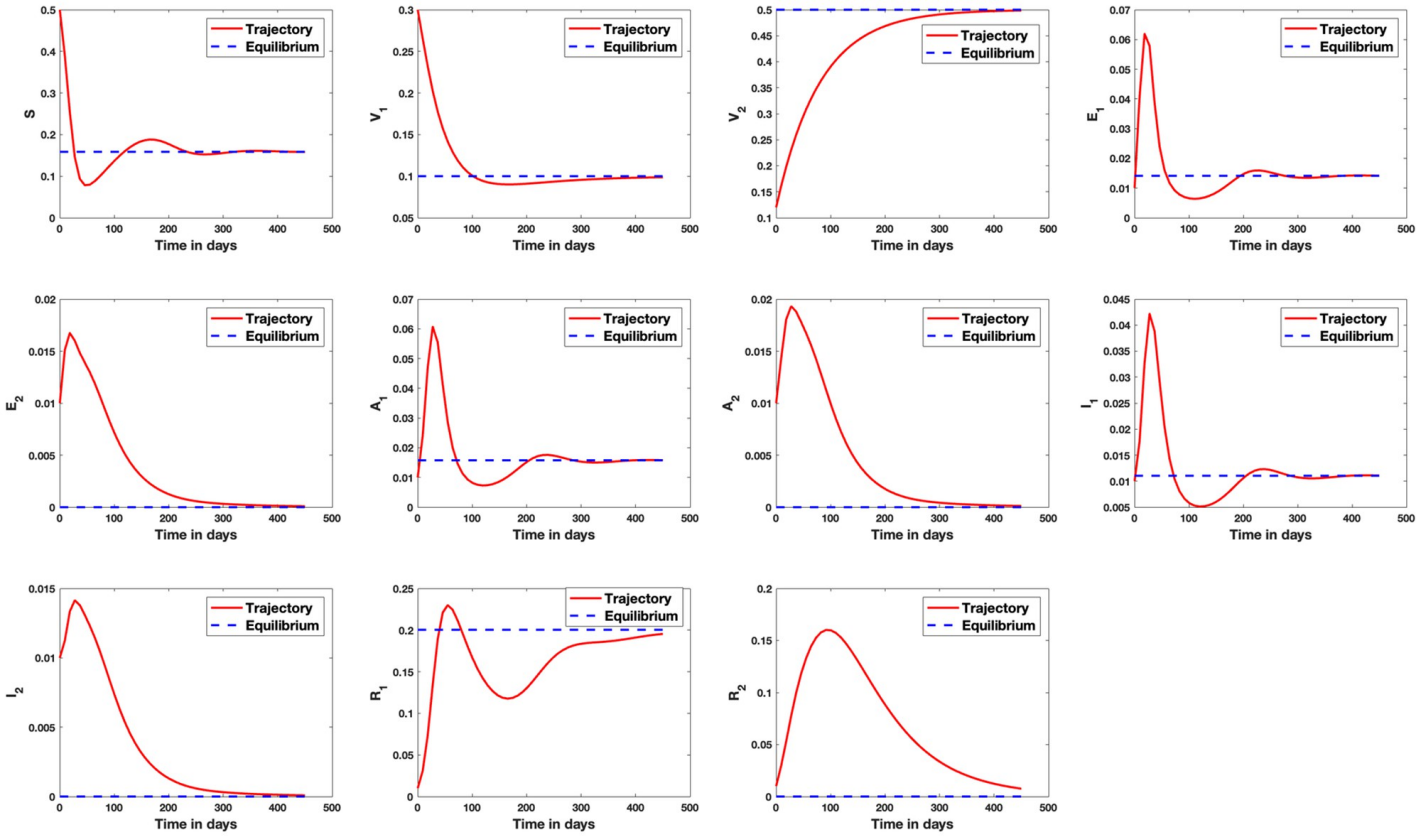
Stability analysis for the case where $R_{1}>R_{2}>1$ but condition (39) is not satisfied. In this case, we set *β*_1_ = 1, *β*_2_ = 0.05, *γ*_1_ = 0.5, *γ*_2_ = 0.4, *μ* = 1/80.3, *q*_1_ = 0.1, *q*_2_ = 0.5, *p* = 0.6, λ_1_ = 0.2, λ_2_ = 0.25, *r*_1_ = 1/10.5, *r*_2_ = 1/9, *θ*_1_ = 0.09, *θ*_2_ = 0.1. The endemic equilibrium in this case is the strain 1 equilibrium $E_{1}=\left\{0.1588,0.1000,0.5000,0.0141,0,0.0158,0,0.0110,0,0.2003,0\right\}$. Here, $R_{1}=2.52$, $R_{2}=1.01$, $1+\frac{R_{1}-1}{1+\frac{Q_{2}-Q_{1}+{\bar{R}}_{1}}{1-q+Q_{1}-{\bar{R}}_{1}}R_{1}}=1.09$ and condition (39) is not satisfied since $R_{1}>1+\frac{R_{1}-1}{1+\frac{Q_{2}-Q_{1}+{\bar{R}}_{1}}{1-q+Q_{1}-{\bar{R}}_{1}}R_{1}}>R_{2}>1$. This condition implies, from (38), that $E_{1}^{+}>0$ and $E_{2}^{+}<0$, so that the values $I_{1}^{+}$, $A_{1}^{+}$, and $R_{1}^{+}$ are positive but $I_{2}^{+}$, $A_{2}^{+}$, and $R_{2}^{+}$ are negative. This shows that only strain 1 endemic exists on the long run. A similar analysis is presented in Appendix B in S1 Appendix geometrically.

**Fig 8:**
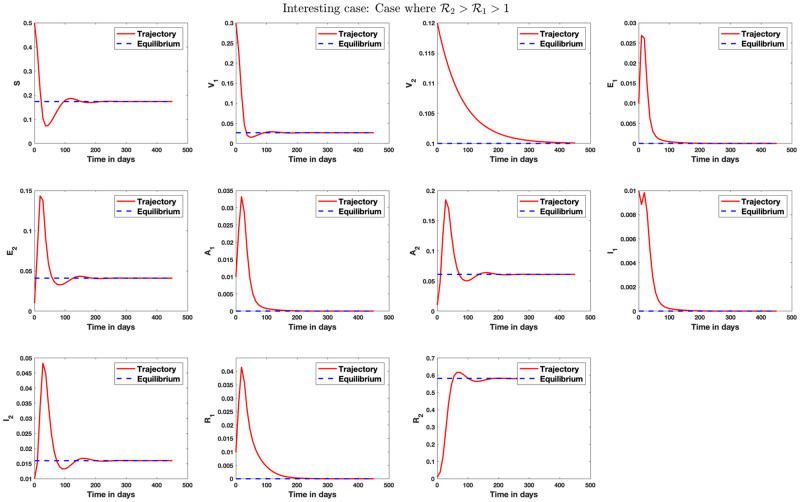
Stability analysis for the case where $R_{2}>R_{1}>1$. In this case, we set *β*_1_ = 0.9, *β*_2_ = 1.0, *γ*_1_ = 0.4, *γ*_2_ = 0.45, *μ* = 1/80.3, *q*_1_ = 0.12, *q*_2_ = 0.1, *p* = 0.8, λ_1_ = 0.21, λ_2_ = 0.2, *r*_1_ = 1/9, *r*_2_ = 1/10.5, *θ*_1_ = 0.1, *θ*_2_ = 0.09. Here, $R_{1}=3.09$ and $R_{2}=4.49$ so that $R_{0}=4.49$. The endemic equilibrium in this case is {0.1739, 0.0267, 0.1000, 0, 0.0410, 0, 0.0609, 0, 0.0160, 0, 0.5815}.

Fig 4 shows simulation result for the case where only strain 1 endemic exists in the population. Similarly, Fig 5 shows simulation result where only strain 2 endemic exists in the population. In Fig 6, we answer the question as to whether it is possible to have an endemic with more than one strain of the virus. The figure shows that this is possible if condition (39) is satisfied. Fig 7 shows the importance of condition (39) by confirming that no two strains remain in the population on the long run even if $R_{1}>R_{2}>1$ but $R_{2}<1+\frac{R_{1}-1}{1+\frac{Q_{2}-Q_{1}+{\bar{R}}_{1}}{1-q+Q_{1}-{\bar{R}}_{1}}R_{1}}$. Fig 8 shows the simulation result for the case where $R_{2}>R_{1}>1$.

#### 5.1.1 What happens if $R_{1}>R_{2}>1$ but condition (39) is not satisfied?

We study a case where $R_{1}>R_{2}>1$ but condition (39) is not satisfied. Although the condition is similar to that in Fig 6, we see here that the system converges to the strain 1 equilibrium point. That is, even though the reproduction numbers $R_{1}$ and $R_{2}$ are more than one, strain 2 still gets eradicated from the system on the long run while strain 1 caused an endemic. This study shows that the second inequality in condition (39) is necessary for the existence of endemic with more than one strain.

#### 5.1.2 What happens if $R_{2}>R_{1}>1$

We study an interesting case where although the reproduction number $R_{1}$ for strain 1 is greater than 1, the strain still gets eradicated from the system on the long run. This happens because a newer strain 2 caused an endemic with a higher reproduction number $R_{2}>R_{1}>1$. This case is analyzed geometrically in Appendix B in S1 Appendix.

### 5.2 Real data: Analysis using the Delta variant cases for the period 10.09.2021 to 12.18.2021

**Fig 9:**
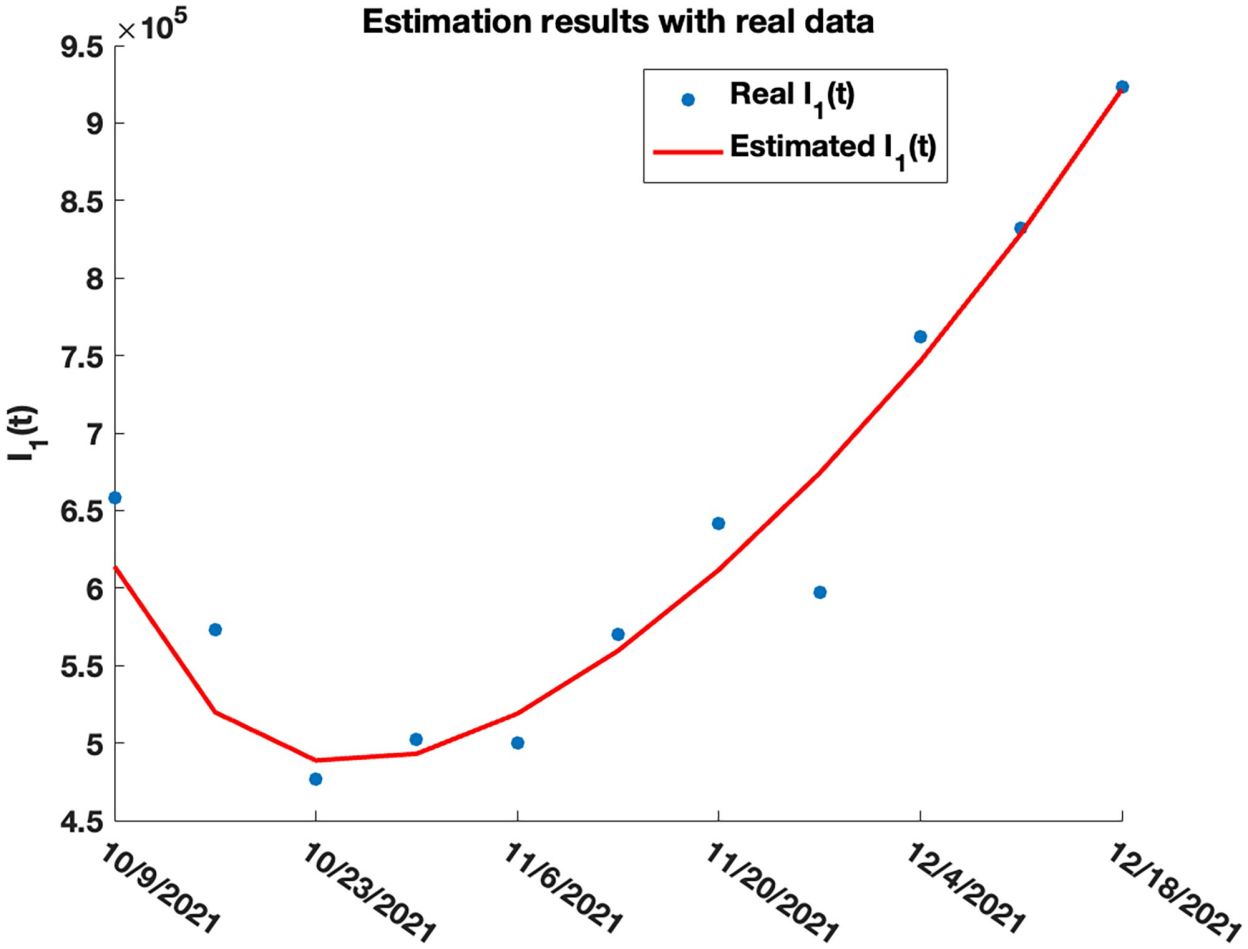
Real (blue) and estimated (red) COVID-19 weekly cases for the Delta variant in the United States.

The work done here is applied to real data using the overall SARS-CoV-2 weekly Variant Proportions [68] for the United States collected from the Centers for Disease Control and Prevention (CDC) for the period 10.09.2021 to 12.18.2021. The proportion of the Delta (B.1.617.2) variant is collected and used together with the number of daily cases (collected from CDC [58]) of all variants in the United States. Within this period, data shows that on the average, the proportion of the Delta variant is about 95% of the total cases in the United State, suggesting that the Delta variant is the dominating variant during the period. For this reason, we set *n* = 1 in (35) and estimated the parameters in Table 4 using the Nelder-Mead simplex algorithm as described in Lagarias et al. [69]. These parameters are estimated by minimizing the sum of square error between the real Delta infection cases and the simulated cases, where the real Delta infection case is generated by multiplying the weekly proportion of the Delta variant by the weekly COVID-19 cases (generated from the daily cases [58]). We use *N* = 331893745 for the total population of the United States, with initial point ${\tilde{y}}_{0}=(S_{0},V_{10},E_{10},A_{10},I_{10},R_{10})=(0.9076,0.0125,0.0001,0.0001,0.0019,0.07)\times N$, and endemic equilibrium point $E_{1}=(0.5108,0.0104,0.2762,0.0351,0.1237,0.0439)\times N$. The real and estimated weekly COVID-19 cases for the United States for period 10.09.2021 to 12.18.2021 are given in Fig 9 and generated using model (35).

**Table 4:** Parameter estimates for model (35) with *n* = 1.

Parameter	${\hat{\beta }}_{1}$	${\hat{\gamma }}_{1}$	$\hat{\mu }$	${\hat{q}}_{1}$	$\hat{p}$	${\hat{\lambda }}_{1}$	${\hat{r}}_{1}$	${\hat{\theta }}_{1}$
Estimate	1.3800	0.6000	0.2045	0.01037	0.3	0.1500	0.1500	0.0300

**Fig 10:**
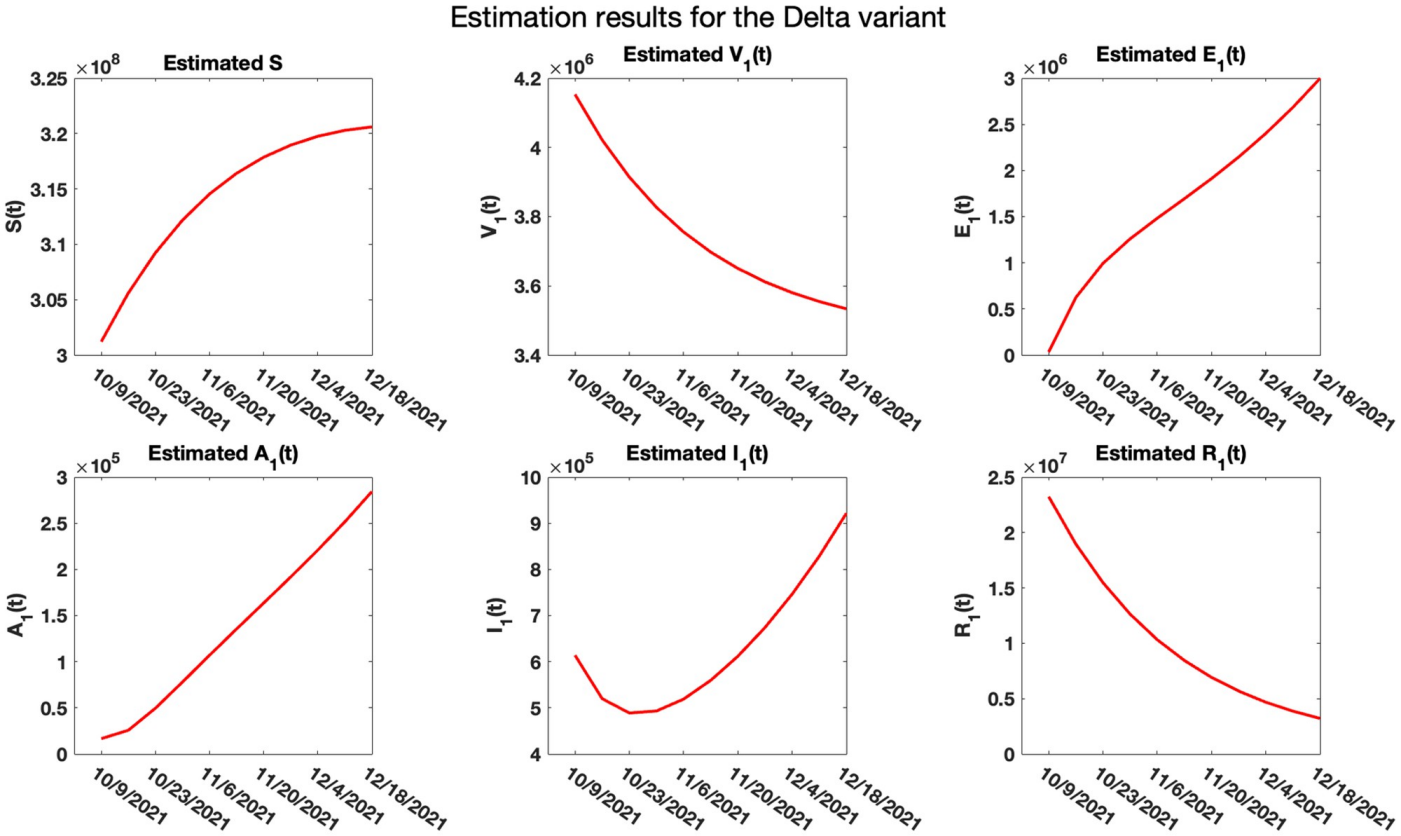
Estimated trajectory path for *S*, *V*_1_, *E*_1_, *A*_1_, *I*_1_, and *R*_1_ for the Delta variant. The analysis suggests that the trajectories of the population of those receiving vaccination against the delta variant and those recovering from the variant are decreasing, while the susceptible, exposed and asymptomatic populations are increasing in the time period 10.09.2021 to 12.18.2021. The symptomatic population decreases from 10.09.2021 to 10.23.2021, after which it started increasing until the end of the analysis in 12.18.2021. The reproduction number $R_{0}$ for the variant, using (14), was calculated to be 1.94.

Fig 10 shows the estimated results for the population sizes *S*, *V*_1_, *E*_1_, *A*_1_, *I*_1_, and *R*_1_ for the Delta variant.

**Fig 11:**
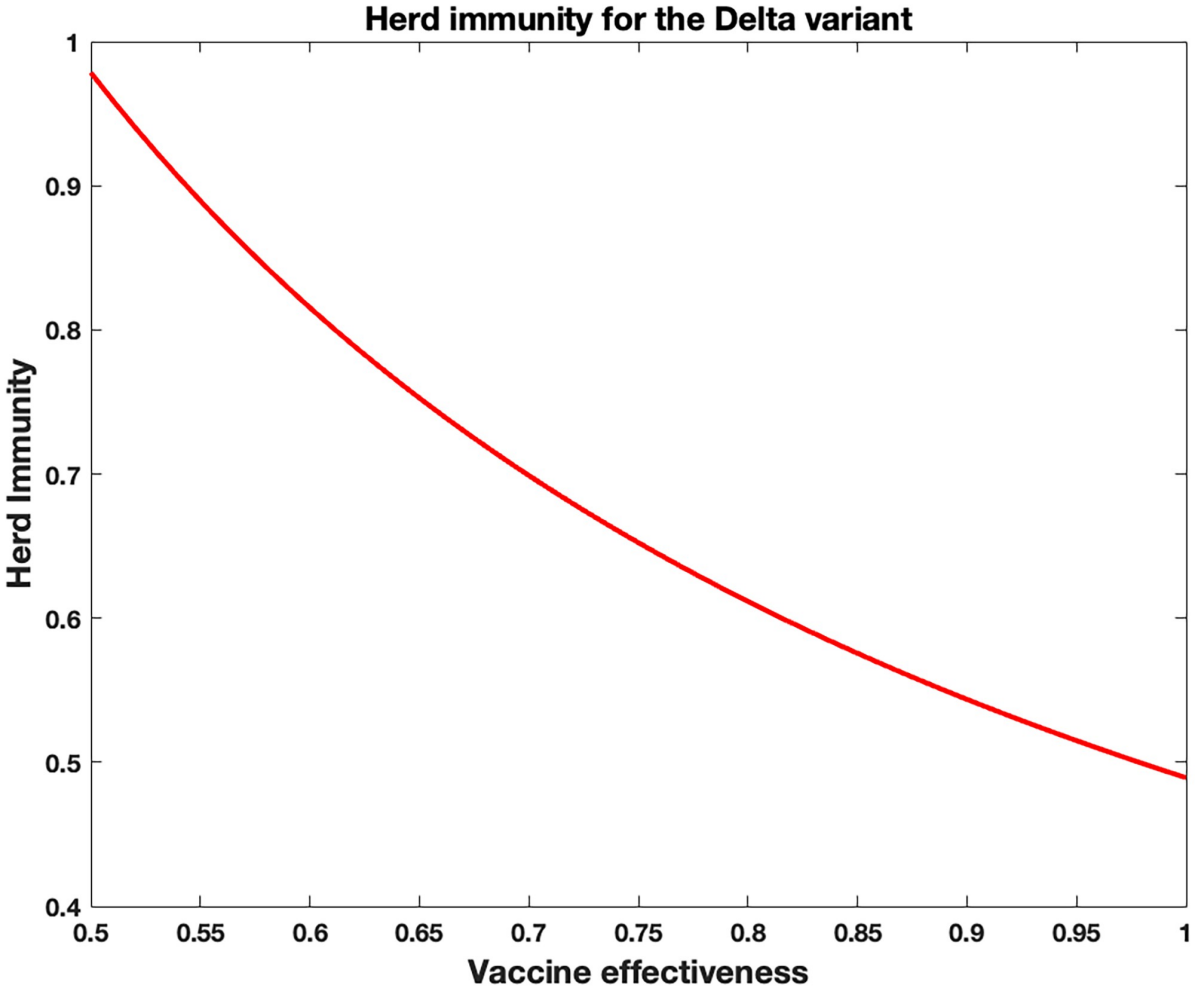
Herd immunity as a function of the measure of the effectiveness of vaccines for the Delta variant in the United States.

Using 88% and 70% for the vaccine effectiveness of two doses of Pfizer vaccine among those with Delta and Omicron variants, we calculate the Herd Immunity threshold bound to be [97%, 1). We give a plot of the Herd immunity as a function of the measure of the effectiveness of available vaccines in Fig 11.

### 5.3 Real data: Analysis using the Delta and Omicron variant cases for the period 12.11.2021 to 01.15.2022

**Fig 12:**
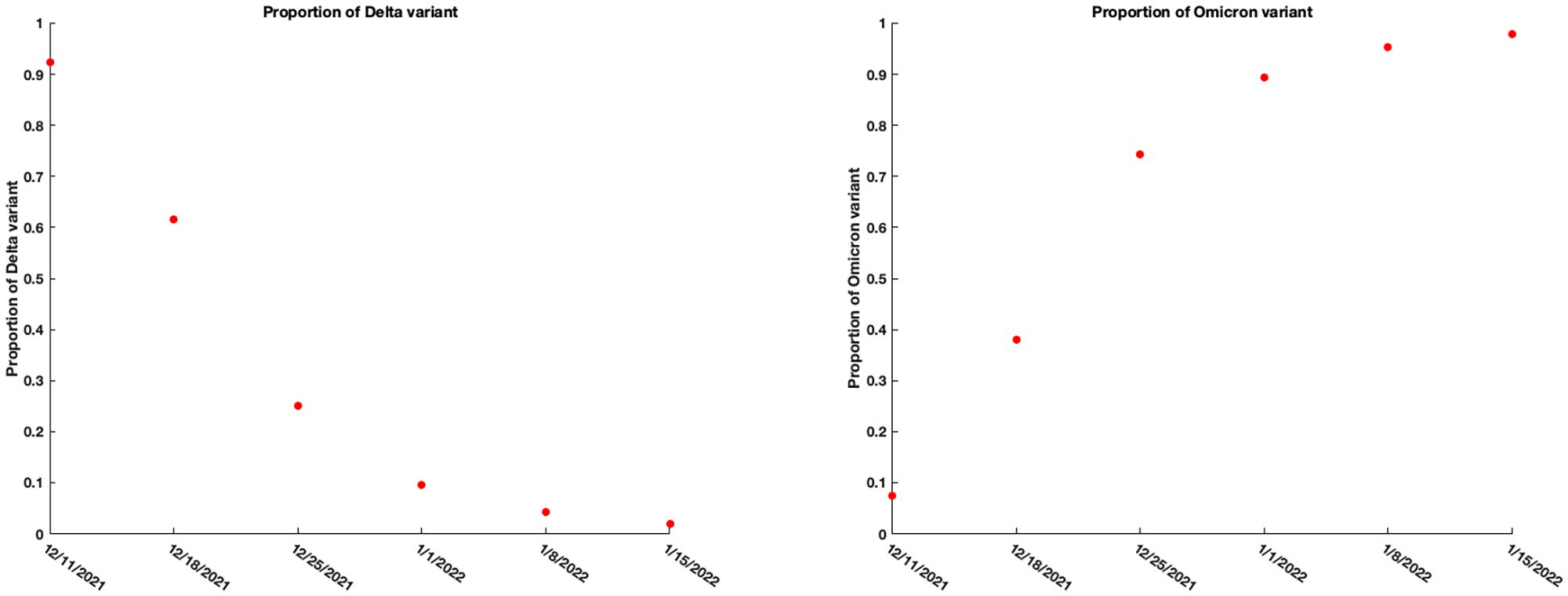
Proportion of the Delta (a) and the Omicron (b) variant for the period 12.11.2021 to 01.15.2022 in the United States collected from CDC2.

**Fig 13:**
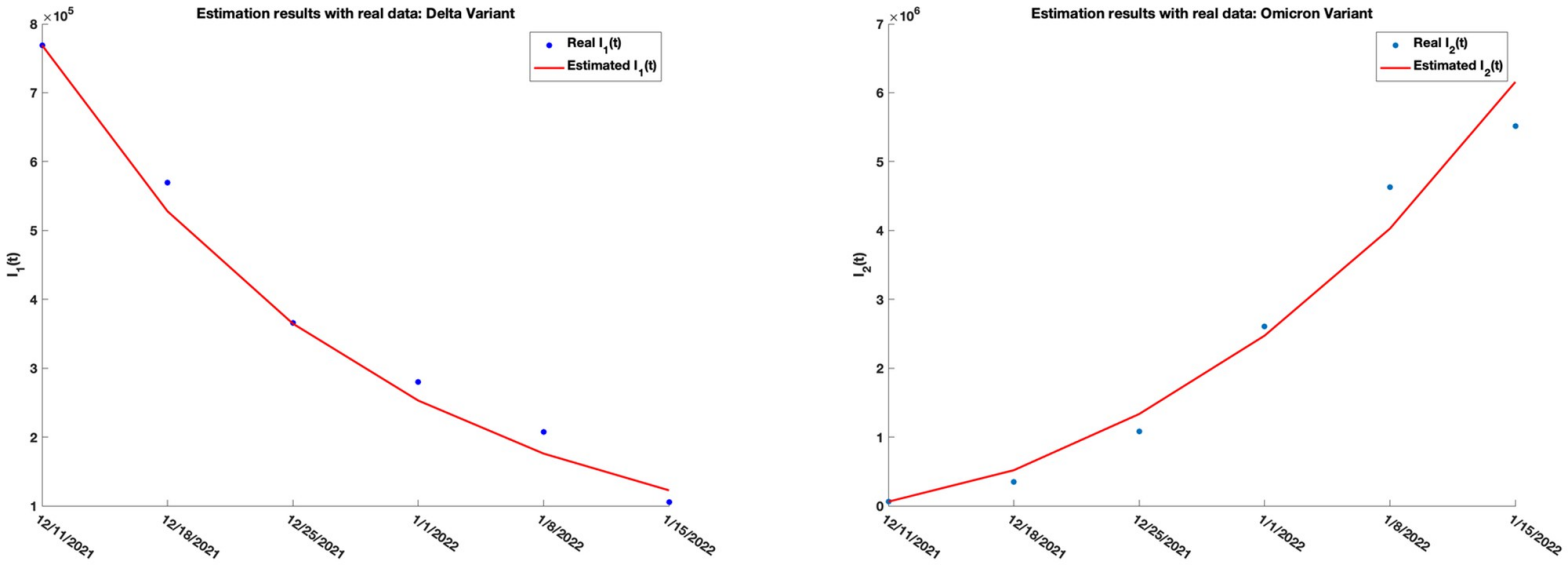
Real (dotted blue) and estimated (red) COVID-19 weekly cases for the Delta variant and the Omicron variant for the period 11.27.2021 to 01.15.2022 in the United States.

The World Health Organization (WHO) and CDC classified a new variant, B.1.1.529, as a VOC and named it Omicron. The work done here is applied to real data using the SARS-CoV-2 weekly Variant Proportions [68] for the United States collected by CDC. The proportion of the Delta (B.1.617.2) and the Omicron (B.1.1.529) variants shown in Fig 12 are collected and used together with the number of weekly cases (collected from CDC [58]) of each variants in the United States. Data suggests the existence of the two variants as VOC and that the infectivity of the Delta variant is slowing down while the infectivity of the Omicron variant is increasing during the period 12.11.2021 to 01.15.2022. For this reason, we set *n* = 2 in (35) and estimated the parameters ${\hat{\beta }}_{1}$, ${\hat{\beta }}_{2}$, ${\hat{\gamma }}_{1}$, ${\hat{\gamma }}_{2}$, $\hat{\mu }$, ${\hat{q}}_{1}$, ${\hat{q}}_{2}$, $\hat{p}$, ${\hat{\lambda }}_{1}$, ${\hat{\lambda }}_{2}$, ${\hat{r}}_{1}$, ${\hat{r}}_{2}$, ${\hat{\theta }}_{1}$, and ${\hat{\theta }}_{2}$ in Table 5 using the Nelder-Mead simplex algorithm [69]. The Delta and Omicron infected data are generated by multiplying the weekly proportion of the Delta variant by the weekly COVID-19 cases. We use *N* = 331893745 for the total population of the United States, with initial point ${\tilde{y}}_{0}=(S_{0},V_{10},V_{20},E_{10},E_{20},A_{10},A_{20},I_{10},I_{20},R_{10},R_{20})=(0.6000,0.0187,0.0014,0.0010,0.0050,0.0010,0.0050,0.0023,0.0002,0.0070,0.0070)\times N$. The real and estimated weekly COVID-19 cases for the infected cases *I*_1_ and *I*_2_ for period 12.11.2021 to 01.15.2022 are given in Fig 13 and generated using model (35). The reproduction number $R_{1}=0.0676$ for the delta variant is less than one. On the other hand, the infectivity of the Omicron variant in this period was so high that the reproduction number obtained was $R_{2}=5.9503$. The strain 2 endemic equilibrium point $E_{1}=(0.1411,0.0018,0.1500,0,0.2864,0,0.0830,0,0.1274,0,0.2104)\times N$ suggests that the Delta variant’s infectivity and exposure will die out on the long run, while there is an Omicron variant endemic. The proportion of the population vaccinated against the delta variant converges to 0.18% on the long run, and that of the Omicron variant converges to 15% on the long run. The proportion of the susceptible, exposed, asymptomatic infected, symptomatic infected, and recovered Omicron population converge to 14.1%, 28.64%, 8.30%, 12.74%, and 21.04% of the population size, respectively, on the long run.

**Table 5:** Parameter estimates for model (35) for the Delta and Omicron variants with *n* = 2.

Parameter	${\hat{\beta }}_{1}$	${\hat{\beta }}_{2}$	${\hat{\gamma }}_{1}$	${\hat{\gamma }}_{2}$	$\hat{\mu }$	${\hat{q}}_{1}$	${\hat{q}}_{2}$	$\hat{p}$	${\hat{\lambda }}_{1}$	${\hat{\lambda }}_{2}$	${\hat{r}}_{1}$	${\hat{r}}_{2}$	${\hat{\theta }}_{1}$	${\hat{\theta }}_{2}$
Estimate	0.0397	2.1614	0.2157	5.6282	0.1500	0.0105	0.1500	0.3946	0.2550	0.2204	0.9987	0.1500	0.2972	0.1500

**Fig 14:**
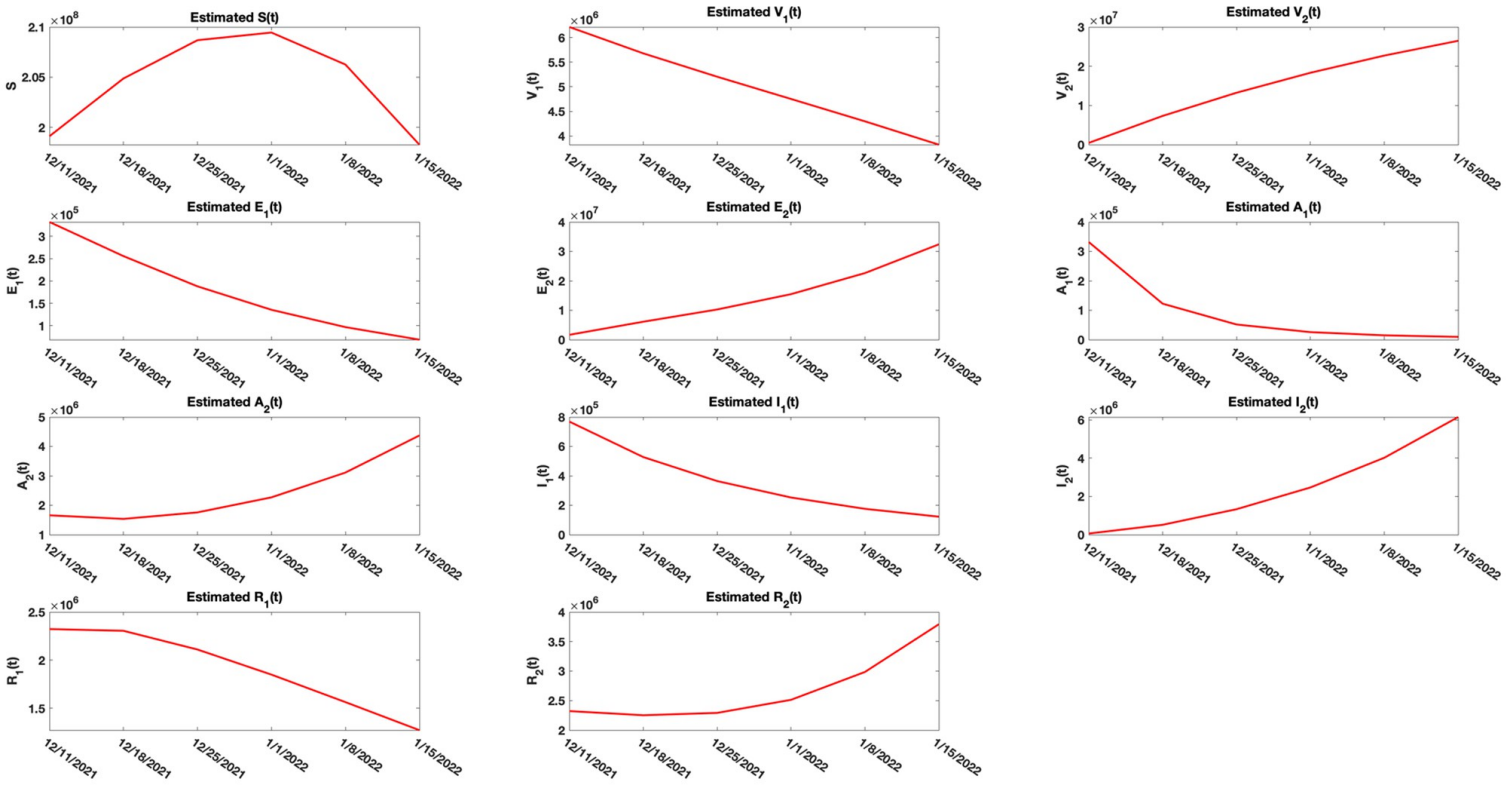
Estimated results for the Delta and Omicron variants. The result shows that the number of Delta exposure and infection cases is decreasing (slowing down) while the number of Omicron exposure and infection cases is increasing (speeding up) between December 11, 2021 and January 15, 2022. These bring about a decrease in the population of those who are vaccinated against the Delta variant and an increase (at a slowing pace) in the number of those vaccinated against the Omicron variant. The number of those susceptible to the variants rises between the period 12.11.2021 to 01.01.2022, after which it started falling until 01.15.2022. The population of those who recovered from the Delta variant is speedingly descreasing while the count of those who recovered from Omicron variant is increasing at a speeding pace. The result of the trajectory of the estimated Delta and Omicron cases is similar to the plot shown in the work of Nyberg et al. [70].

The estimated results for all compartments are shown in Fig 14.

#### 5.3.1 Sensitivity analysis

In this section, we study the impact of each epidemiological parameters on the disease transmission and prevalence. We are interested in discovering the parameters that have high impact on the basic reproduction number $R_{k}$, *k* = 1, 2, ⋯, *n*. This can be achieved using sensitivity analysis by calculating the sensitivity index of each parameters on $R_{k}$. The normalized forward sensitivity index $\Delta_{p}^{u}$ of a variable *F* that depends differentiably on a parameter *u* is defined as [71, 72]

$$\begin{array}{c} \Delta_{u}^{F}=\frac{\partial F}{\partial u}\cdot \frac{u}{F}. \end{array}$$

The analytic expression for the sensitivity index of $R_{k}$, *k* = 1, 2, ⋯, *n*, with respect to the parameters in (35) is obtained as

$$\begin{array}{ccc} \Delta_{\beta_{k}}^{R_{k}} & = & \frac{\left(1-p\right)a_{k}\beta_{k}}{c_{k}},\\ \Delta_{\gamma_{k}}^{R_{k}} & = & \frac{pw_{k}\gamma_{k}}{c_{k}},\\ \Delta_{\mu }^{R_{k}} & = & -\frac{1}{c_{k}}(\left(1-p\right)a_{k}\beta_{k}(\frac{1}{e_{k}}+\frac{1}{w_{k}})+pw_{k}\gamma_{k}(\frac{1}{e_{k}}+\frac{1}{a_{k}})),\\ \Delta_{q_{j}}^{R_{k}} & = & 0,\;j=1,2,\cdots ,k-1,\\ \Delta_{q_{k+j}}^{R_{k}} & = & -\frac{1}{1-q+Q_{k}},\;j=0,1,\cdots ,n-k,\\ \Delta_{p}^{R_{k}} & = & \frac{a_{k}w_{k}}{c_{k}}(\frac{\gamma_{k}}{a_{k}}-\frac{\beta_{k}}{w_{k}}),\\ \Delta_{\lambda_{k}}^{R_{k}} & = & \frac{\mu }{\lambda_{k}e_{k}},\\ \Delta_{r_{k}}^{R_{k}} & = & -\frac{pw_{k}\gamma_{k}}{a_{k}c_{k}},\\ \Delta_{\theta_{k}}^{R_{k}} & = & -\frac{\left(1-p\right)a_{k}\beta_{k}}{w_{k}c_{k}}. \end{array}$$

The positive sensitivity index with respect to *β*_*k*_, *γ*_*k*_, and λ_*k*_ shows that an increase in the value of the strain *k*’s symptomatic, asymptomatic transmission rates, and the latency rate leads to an increase in the basic reproduction number, $R_{k}$, as expected. Likewise, the negative sensitivity index for *μ*, *q*_*l*_, *l* = *k*, *k* + 1, ⋯, *n*, *r*_*k*_, and *θ*_*k*_ shows that an increase in the natural death rate, vaccination rate, symptomatic and asymptomatic recovery rates, respectively, leads to a decrease in $R_{k}$, as expected. Also, the zero value for the sensitivity index for *q*_*l*_, *l* = 1, 2, ⋯, *k* − 1, shows that being vaccinated against predecessor strains *l* = 1, 2, ⋯, *k* − 1 does not have any impact on the current and future strains *j* ≥ *k* infection count. The sensitivity index $\Delta_{p}^{R_{k}}$ is positive if $\frac{\gamma_{k}}{a_{k}}>\frac{\beta_{k}}{w_{k}}$. That is, an increase in the fraction of infection cases that are asymptomatic will lead to an increase in the basic reproduction number, $R_{k}$, if the total number of infection caused by an asymptomatic strain *k* infectious individual is more than that caused by a symptomatic strain *k* individual. The magnitude of the sensitivity of $R_{k}$ to changes in these parameters can be calculated by evaluating $\Delta_{u}^{R_{k}}$ for each parameter estimates *u* in Tables 4 and 5 for case where *n* = 1 and *n* = 2, respectively. For a particular parameter *u*, a higher value of $\Delta_{u}^{R_{k}}$ suggests that $R_{k}$ is more sensitive to *u*. The sensitivity index of $R_{k}$ to each parameters estimated in Table 5 for model (35) is given in Table 6 for *k* = 1, 2.

**Table 6:** Sensitivity index of $R_{k}$ to parameters generated in Table 5 for model (35).

	Parameters *u*							
	$\Delta_{u}^{R_{k}}$	*β*_*k*_	*γ*_*k*_	*μ*	*q*_*k*_	*p*_*k*_	λ_*k*_	*r*_*k*_	*θ*_*k*_
*k* = 1	$\Delta_{u}^{R_{1}}$	0.4204	0.5796	−3.9138	−1.1912	0.7744	1.4524	−0.5046	−0.9401
*k* = 2	$\Delta_{u}^{R_{2}}$	0.3707	0.6293	−5.0312	−1.1765	0.9823	1.8374	−2.0975	−1.2358

Analysis suggests that changes in the asymptomatic transmission rate contribute more to the changes in the reproduction number $R_{k}$ than the symptomatic transmission rate for the Delta (*k* = 1) and Omicron (*k* = 2) variants. Also, $R_{k}$ is more sensitive to changes in the infection rate λ_*k*_ than the transmission rates and the fraction *p*_*k*_ of cases that are asymptomatic. For the Delta variant, the reproduction number $R_{1}$ is more sensitive to the recovery rate *θ*_1_ of the symptomatic class than *r*_1_. Likewise for the Omicron variant, the asymptomatic recovery rate *r*_2_ has much influence over $R_{2}$ than the symptomatic recovery rate *θ*_2_.

## 6 Summary and discussion

In this work, a multi-variant epidemic model for analyzing the emergence and dissemination of viral multi-strains of an infectious disease in a population that is assumed to be completely susceptible to *n* different strains of the disease is developed and analyzed. A major assumption on the viral strains is that those who are vaccinated and recovered from a specific strain *k* ≤ *n* are immune to that strain and its predecessors but can still be infected by newer emerging strains. A study of how well-poised the model is is carried out by showing the existence of non-negative solution of the derived model. The model compares the cases where the force of infection on the susceptible vaccinated and unvaccinated populations are different and the same. The reproduction number for each specific strains *j* = 1, 2, ⋯, *n* is obtained and analyzed. We show that the reproduction number for the system where individuals who recovered from certain strain can be infected by emerging strain is the same for the system where such individuals cannot be re-infected by emerging strain. In order to shed more light on the possibility of an endemic with more than one strain of the virus, global stability analysis is obtained for different equilibrium points $E_{S_{r}}$, *r* = 0, 1, 2, ⋯, *n*, of the system, with $E_{S_{0}}$ denoting the disease-free equilibrium. It was shown with respect to model (35) that for an endemic with strains *τ*_1_ ≤ *n* and *τ*_2_ ≤ *n* (where *τ*_1_ < *τ*_2_) to occur, it must be that the reproduction number $R_{\tau_{1}}>R_{\tau_{2}}>1+\frac{R_{\tau_{1}}-1}{1+\frac{Q_{\tau_{2}}-Q_{\tau_{1}}+{\bar{R}}_{1}}{1-q+Q_{\tau_{1}}-{\bar{R}}_{1}}R_{\tau_{1}}}$. This condition shows that for an endemic with strains *τ*_1_ ≤ *n* and *τ*_2_ ≤ *n* to occur, the population must have been in an endemic state with the first emerged strain *τ*_1_ and the number of secondary infection caused by strain *τ*_1_ must be greater than that of strain *τ*_2_, with a necessary condition that $R_{\tau_{2}}>1+\frac{R_{\tau_{1}}-1}{1+\frac{Q_{\tau_{2}}-Q_{\tau_{1}}+{\bar{R}}_{1}}{1-q+Q_{\tau_{1}}-{\bar{R}}_{1}}R_{\tau_{1}}}$. The importance of this necessary condition is emphasized numerically by showing that a system that only satisfies the condition $R_{\tau_{1}}>R_{\tau_{2}}>1$ does not converge to the endemic equilibrium $E_{S_{2}}$ with strains *τ*_1_ and *τ*_2_, but instead converges to strain *τ*_1_ equilibrium $E_{\tau_{1}}$. The later condition reduces to $R_{\tau_{1}}>R_{\tau_{2}}>1+\frac{R_{\tau_{1}}-1}{1+\frac{Q_{\tau_{2}}-Q_{\tau_{1}}}{1-q+Q_{\tau_{1}}}R_{\tau_{1}}}$ for the case where individuals who recovered from certain strain cannot be infected by emerging strain. A general condition is obtained for the case where endemic with strains *τ*_1_, *τ*_2_, ⋯, *τ*_*r*_ (with *τ*_1_ < *τ*_2_ < ⋯ < *τ*_*r*_) exists in the population. Our analysis shows that for an endemic with strains *τ*_1_, *τ*_2_, ⋯, *τ*_*r*_ to exist, the population must first be in endemic with strain *τ*_1_, followed by *τ*_2_, ⋯, *τ*_*r*_, so that condition (24) satisfied. Also, we showed numerically that a strain with a reproduction number greater than 1 can still die out on the long run if a newer emerging strain has a greater reproduction number. The effect of vaccines on the population is also analyzed. The herd immunity for each strain *j* = 1, 2, ⋯, *n* is computed as a function of the effectiveness of vaccines against the strain. A threshold for the herd immunity for the case where there is endemic of more than one strain of the virus is also calculated. The result is applied to analyze real COVID-19 data for the Delta and Omicron variants in the United States. The reproduction numbers for the Delta and Omicron variants cases for the period 12.11.2021 to 01.15.2022 suggest that the Delta variant cases is dying down, while there is an endemic of the Omicron variant. Using model (35), possible trajectories for the susceptible, vaccinated, exposed, infected and recovered population are plotted by first estimating the parameters in the model. The parameters are estimated by minimizing the sum of square error between the real and estimated infection cases using the Nelder-Mead simplex algorithm [69]. Further research on the COVID-19 cases is ongoing and the results of the research will be made known once it is available. In the future, we plan to modify the model to fit emerging characteristics of the virus and extends limitations in the model to a more general case.

## Supporting information

S1 Appendix (PDF)
