# Supplementary material for: Analysis of multi-strain infection of vaccinated and recovered population through epidemic model: Application to COVID-19
Source: PLoS One. 2022 Jul 29;17(7):e0271446. doi: 10.1371/journal.pone.0271446 (PMC9337708; doi:10.1371/journal.pone.0271446)
Supplement: S1 Appendix — (PDF) [file pone.0271446.s001.pdf]

A Proof of Existence of $\mathcal{E}_m$ for model (1) for the case where $\beta_k \neq \beta_{k,j}$ and $\gamma_k \neq \gamma_{k,j}$

Lemma 17. *Let* $\kappa_m = \min\limits_{2\leq j\leq m}\{\hat{c}_{m,j-1}, c_m\}$. *The function*

$$f(z) = \frac{(1-q)\mu c_m\lambda_m}{\mu + \frac{c_m\lambda_m}{a_m w_m}z} + \sum_{j=1}^{m}\left(\frac{q_{j-1}\mu\hat{c}_{m,j-1}\lambda_m}{\mu + \frac{\hat{c}_{m,j-1}\lambda_m}{a_m w_m}z}\right) - a_m e_m w_m$$

has a zero in the interval $\left(0, \frac{a_m w_m \mu}{\lambda_m \kappa_m}\mathcal{R}_m\right)$ *if* $\mathcal{R}_m > 1$.

Proof. The function $f(z)$ is clearly continuous on $\left[0, \frac{a_m w_m \mu}{\lambda_m \kappa_m}\mathcal{R}_m\right]$ and

$$f(0) \;=\; (1-q)c_m\lambda_m + \sum_{j=1}^{m}(q_{j-1}\hat{c}_{m,j-1}\lambda_m) - a_m e_m w_m = a_m e_m w_m(\mathcal{R}_m - 1) > 0$$

if $\mathcal{R}_m > 1$. Also,using Remark 1, we have

$$\begin{aligned} f\left(\frac{a_m w_m \mu}{\lambda_m \kappa_m}\mathcal{R}_m\right) &= \frac{(1-q)\mu c_m\lambda_m}{\mu + \frac{c_m\mu}{\kappa_m}\mathcal{R}_m} + \sum_{j=1}^{m}\left(\frac{q_{j-1}\mu\hat{c}_{m,j-1}\lambda_m}{\mu + \frac{\hat{c}_{m,j-1}\mu}{\kappa_m}\mathcal{R}_m}\right) - a_m e_m w_m \\ &< \frac{(1-q)c_m\lambda_m + \sum\limits_{j=1}^{m}(q_{j-1}\hat{c}_{m,j-1}\lambda_m)}{\mathcal{R}_m} - a_m e_m w_m = 0. \end{aligned}$$

Hence, it follows from the Intermediate Value Theorem that there exist a number $z_m \in \left(0, \frac{a_m w_m}{\lambda_m \kappa_m}\mathcal{R}_m\right)$ such that $f(z_m) = 0$. ■

Theorem 18. *The strain m unique equilibrium point* $\mathcal{E}_m$ *for the epidemic models (1) and (35) exists in the feasible region* $\mathcal{T}$ *provided* $\mathcal{R}_m > 1$. *Furthermore, the value of* $\mathcal{E}_m$ *is the same for models (1) and (35).*

Proof. The strain m equilibrium point $\mathcal{E}_m$ is obtained as

$$\mathcal{E}_m = \{S^*, V_1^*, \cdots, V_n^*, E_1^*, \cdots, E_n^*, A_1^*, \cdots, A_n^*, I_1^*, \cdots, I_n^*, R_1^*, \cdots, R_n^*\}, \tag{40}$$

where

$$\begin{aligned} S^* &= \tfrac{(1-q)\mu}{\mu + (\beta_m I_m^* + \gamma_m A_m^*)} = \tfrac{(1-q)\mu}{\mu + \frac{c_m\lambda_m}{a_m w_m}E_m^*} \\ V_k^* &= \begin{cases} \frac{q_k\mu}{\mu + \frac{\hat{c}_{m,k}\lambda_m}{a_m w_m}E_m^*}, & \text{if } k < m, \\ q_k, & \text{if } k \geq m, \end{cases} \\ A_m^* &= \tfrac{p\lambda_m}{a_m}E_m^*, \\ I_m^* &= \tfrac{(1-p)\lambda_m}{w_m}E_m^*, \\ R_m^* &= \tfrac{(1-p)a_m\theta_m + p w_m r_m}{\mu a_m w_m}\lambda_m E_m^*, \\ E_k^* &= A_k^* = I_k^* = R_k^* = 0 \;\forall\; k \neq m, \end{aligned} \tag{41}$$

and E_m^* satisfies

$$\frac{c_m\lambda_m}{a_m w_m}S^* + \sum_{j=1}^{m}V_{j-1}^*\left(\frac{\hat{c}_{m,j-1}\lambda_m}{a_m w_m}\right) - e_m = 0, \tag{42}$$

where we set $V_0^*=0$. This value is the same for models (1) and (35). Equation (42) reduces to

$$\frac{(1-q)\mu c_m\lambda_m}{\mu+\frac{c_m\lambda_m}{a_m w_m}E_m^*}+\sum_{j=1}^{m}\left(\frac{q_{j-1}\mu\hat{c}_{m,j-1}\lambda_m}{\mu+\frac{\hat{c}_{m,j-1}\lambda_m}{a_m w_m}E_m^*}\right)-a_m e_m w_m=0. \tag{43}$$

If $\mathcal{R}_m>1$, the existence of E_m^* in the interval $\left(0,\frac{a_m w_m\mu}{\lambda_m\kappa_m}\mathcal{R}_m\right)$ follows from Lemma 17. We can show that $S^*,\{V_k\}_{k=1}^n,E_m^*,A_m^*,I_m^*,R_m^*\in(0,1)$ by using (5) and (42) to show that

$$\begin{aligned}
S^*+E_m^*+A_m^*+I_m^*+R_m^*+\sum_{k=1}^{n}V_k^* &= \frac{(1-q)\mu}{\mu+\frac{c_m\lambda_m}{a_m w_m}E_m^*}+\sum_{k=1}^{m-1}\frac{q_k\mu}{\mu+\frac{\hat{c}_{m,k}\lambda_m}{a_m w_m}E_m^*}+\sum_{k=m+1}^{n}q_k+E_m^*\\
&\quad+\frac{pw_m+(1-p)a_m}{a_m w_m}\lambda_m E_m^*+\frac{pw_m r_m+(1-p)a_m\theta_m}{\mu a_m w_m}\lambda_m E_m^*\\
&= (1-q)+\sum_{k=1}^{n}q_k+E_m^*+\frac{pw_m+(1-p)a_m}{a_m w_m}\lambda_m E_m^*\\
&\quad+\frac{pw_m r_m+(1-p)a_m\theta_m}{\mu a_m w_m}\lambda_m E_m^*-\frac{e_m}{\mu}E_m^*\\
&= 1+\frac{\lambda_m}{\mu}E_m^*+E_m^*-\frac{e_m}{\mu}E_m^*\\
&= 1.
\end{aligned}$$

■

B Geometric analysis of the stability of equilibrium points for the case where $n=2$.

Fig 15 shows a geometric plot of the stability regions for the DFE, strain 1, strain 2 and the endemic $\mathcal{E}_{\mathcal{S}_2}$ equilibrium points.

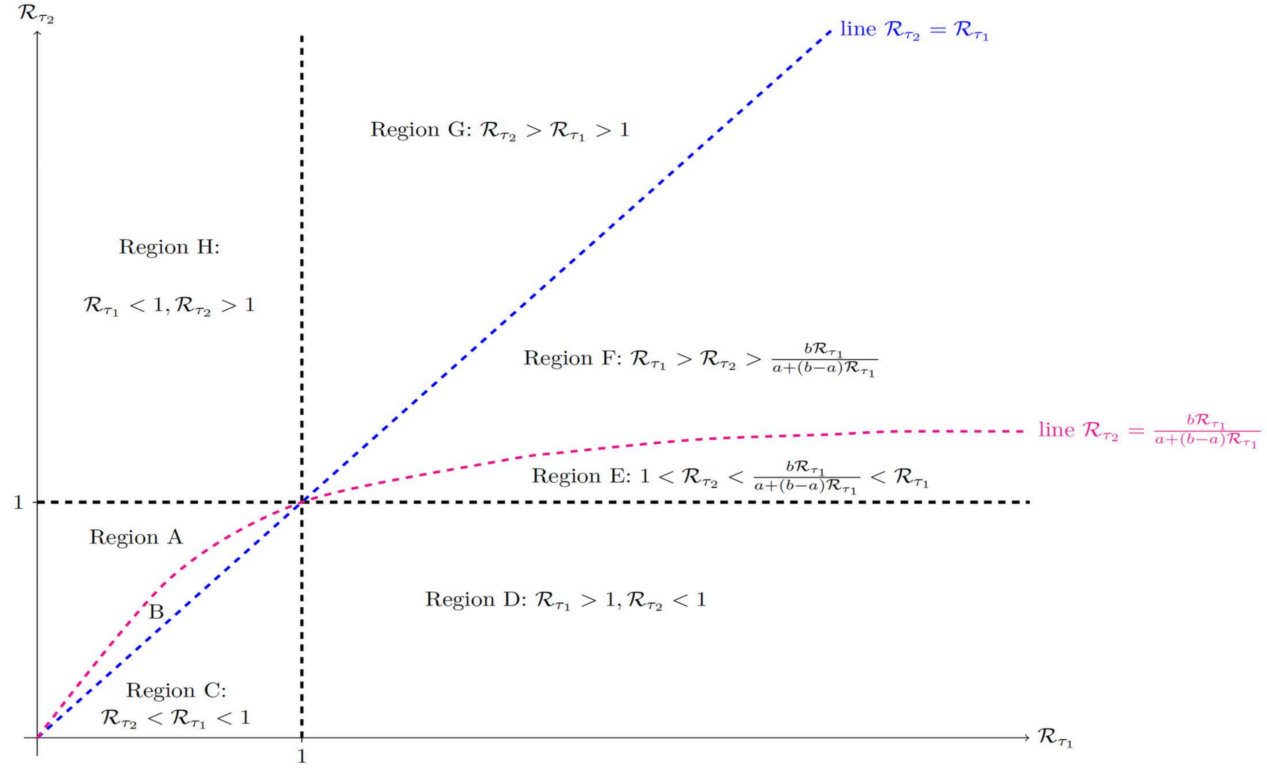

Fig 15. Diagram showing stability regions for the DFE and specific strain τ_k equilibrium. Region A is the region satisfying $\frac{b\mathcal{R}_{\tau_1}}{a+(b-a)\mathcal{R}_{\tau_1}} < \mathcal{R}_{\tau_2} < 1$, where

$$\begin{aligned} a &= 1 - q + Q_{\tau_1}, \text{ and } b = 1 - q + Q_{\tau_2} \text{ for model (1)}, \\ a &= 1 - q + Q_{\tau_1} - \overline{\mathcal{R}}_{\tau_1}, \text{ and } b = 1 - q + Q_{\tau_2} \text{ for model (35)}. \end{aligned}$$

Region B occurs when $\mathcal{R}_{\tau_1} < \mathcal{R}_{\tau_2} < \frac{b\mathcal{R}_{\tau_1}}{a+(b-a)\mathcal{R}_{\tau_1}} < 1$. The DFE is globally stable in the region where $\mathcal{R}_0 = \max\{\mathcal{R}_{\tau_1}, \mathcal{R}_{\tau_2}\} < 1$ (regions A, B, and C fall in this region). The strain τ_1 equilibrium is globally stable in region D. The strain τ_2 equilibrium is globally stable in region H. The endemic equilibrium $\mathcal{E}_{\mathcal{S}_2}$ is globally stable in region F. In region E, we see that $1 < \mathcal{R}_{\tau_2} < \frac{b\mathcal{R}_{\tau_1}}{a+(b-a)\mathcal{R}_{\tau_1}} < \mathcal{R}_{\tau_1}$. This condition implies, from (38), that the compartmental equilibrium value $E^+_{\tau_1} > 0$ and $E^+_{\tau_2} < 0$, so that the values $I^+_{\tau_1}$, $A^+_{\tau_1}$, and $R^+_{\tau_1}$ are positive but $I^+_{\tau_2}$, $A^+_{\tau_2}$, and $R^+_{\tau_2}$ are negative. This shows that only strain τ_1 endemic exists in this region. It can be shown in a similar manner that only one strain remains in the system on the long run if $\mathcal{R}_{\tau_2} > \mathcal{R}_{\tau_1} > 1$ in Region G.
